# The Interaction of Human Pathogenic Fungi With C-Type Lectin Receptors

**DOI:** 10.3389/fimmu.2018.01261

**Published:** 2018-06-04

**Authors:** Surabhi Goyal, Juan Camilo Castrillón-Betancur, Esther Klaile, Hortense Slevogt

**Affiliations:** ^1^Institute for Microbiology and Hygiene, Charité – Universitätsmedizin Berlin, Berlin, Germany; ^2^Septomics Research Center, Jena University Hospital, Jena, Germany; ^3^International Leibniz Research School for Microbial and Biomolecular Interactions, Leibniz Institute for Natural Product Research and Infection Biology/Hans Knöll Institute, Jena, Germany

**Keywords:** pattern recognition receptor, C-type lectin receptor, *Candida*, *Aspergillus*, pathogenic fungi, single nucleotide polymorphisms

## Abstract

Fungi, usually present as commensals, are a major cause of opportunistic infections in immunocompromised patients. Such infections, if not diagnosed or treated properly, can prove fatal. However, in most cases healthy individuals are able to avert the fungal attacks by mounting proper antifungal immune responses. Among the pattern recognition receptors (PRRs), C-type lectin receptors (CLRs) are the major players in antifungal immunity. CLRs can recognize carbohydrate ligands, such as β-glucans and mannans, which are mainly found on fungal cell surfaces. They induce proinflammatory immune reactions, including phagocytosis, oxidative burst, cytokine, and chemokine production from innate effector cells, as well as activation of adaptive immunity *via* Th17 responses. CLRs such as Dectin-1, Dectin-2, Mincle, mannose receptor (MR), and DC-SIGN can recognize many disease-causing fungi and also collaborate with each other as well as other PRRs in mounting a fungi-specific immune response. Mutations in these receptors affect the host response and have been linked to a higher risk in contracting fungal infections. This review focuses on how CLRs on various immune cells orchestrate the antifungal response and on the contribution of single nucleotide polymorphisms in these receptors toward the risk of developing such infections.

## Introduction

Fungi are ubiquitously present in the environment and as commensals in humans; therefore, innate immunity needs to continuously work against the constant exposure. Pattern recognition receptors (PRRs) found on cell surfaces and as soluble forms in body fluids can recognize microbe-specific molecules; the so-called pathogen-associated molecular patterns (PAMPs). PRRs are expressed on immune cells and also on epithelial cells. The interaction of PRRs with PAMPs induces cell- and receptor-specific cellular host responses involving both, the innate and the acquired immune system. There are mainly four different kinds of PRR families, including the toll-like receptors, Nod-like receptors, C-type lectin receptors (CLRs), and RIG-I-like receptors (1). CLRs can recognize carbohydrates by virtue of having a C-type lectin-like domain (2). The domain consists of a conserved double loop structure and a long, structurally, and evolutionarily flexible loop which is involved in Ca^2+^-dependent carbohydrate binding (3). The characteristic fungal cell wall feature is its richness in carbohydrates and, therefore, they serve as the candidate targets for recognition by CLRs. It is widely accepted that CLRs play major role in antifungal immunity compared to other PRRs (1, 4).

C-type lectin receptors can be found as soluble forms in the serum and other body fluids or as transmembrane receptors on various immune cells, such as macrophages, dendritic cells (DCs), neutrophils, and various other cell types (Table 1) (5). Although CLRs have been divided based on their domain organization and phylogenetic features (3, 6), a broader classification of transmembrane receptors is possible based on the type of signaling mechanisms employed by them (7). One such mechanism is the signaling of CLRs *via* immunoreceptor tyrosine-based activation motifs (ITAMs). The ITAM motif (consensus sequence YxxL/I) recruits and phosphorylates Syk kinase on receptor ligation. Signaling *via* Syk typically leads to NF-κB activation *via* the complex consisting of caspase recruitment domain-containing protein 9 (CARD9) singalosome, a trimeric CARD9, B cell lymphoma/leukemia 10, and the mucosa-associated lymphoid tissue lymphoma translocation protein 1. Syk activation ultimately induces subsequent proinflammatory responses, as well as other responses, such as phagocytosis and reactive oxygen species (ROS) and reactive nitrogen species (RNS) production (8, 9). Some CLRs do not have their own cytoplasmic ITAMs. Such receptors couple with ITAM containing adaptor molecules like FcRγ to emanate signaling (10, 11). Dectin-1 is another non-classical CLR bearing a hemITAM motif (consensus sequence YxxL) and the ligand binding is Ca^2+^ independent (12). A second signaling mechanism with contrary effects to those elicited by ITAM signaling is employed by CLRs containing a cytoplasmic immunoreceptor tyrosine-based inhibitory motif (ITIM). Here, receptor ligation leads to the phosphorylation of tyrosine within the ITIM motif (consensus sequence I/V/L/SxYxxI/L/V) and the recruitment of SHP-1, SHP-2, and/or SHIP-1 phosphatases which exert an inhibitory effect by dampening the proinflammatory response (13, 14). Finally, some CLRs do not contain any known signaling motifs and, therefore, only little is known about their signaling mechanisms, such as LOX-1, MR, and langerin.

**Table 1:** C-type lectin receptors and their respective ligands involved in fungal recognition by different human cell types.

Receptor	Fungus	Ligand	Cell Type	Reference
Dectin-1	*Aspergillus fumigatus, Malassezia* spp., *Saccharomyces cerevisiae, Fonsecaea pedrosoi, Pneumocystis carinii, Cryptococcus neoformans, Paracoccidioides brasiliensis, Histoplasma capsulatum, Coccidioides posadasii, T. mentagrophytes, Candida albicans, Talaromyces marneffei, Fusarium solani, Exserohilum rostratum, Cladosporium cladosporioides, T. asahii*, and *Sporothrix schenckii*	β-1,3-glucan	hDC, macrophages, bronchial epithelial cells, monocytes, neutrophils, mast cells, dendritic cells (DCs), and pulmonary epithelium	(12, 15–32)

Dectin-2	*A. fumigatus, Malassezia* spp., *F. pedrosoi, H. capsulatum, T. rubrum, C. albicans, C. glabrata*, and *M. audouinii*	α-mannans	DCs, macrophages	(11, 33–37)

MR	*A. fumigatus, S. cerevisiae, P. carinii, C. neoformans, P. brasiliensis, C. albicans, C. parapsilosis, T. marneffei*, and *S. schenckii*	gp43 (*P. brasiliensis*), mannoproteins (*C. neoformans*), and α-mannans	Corneal epithelial cells, alveolar macrophages (AMs), DCs, monocytes, and keratinocytes	(38–47)

SP-A, SP-D	*A. fumigatus, S. cerevisiae, Pneumocystis* spp., *C. neoformans, Histoplasma* spp., and *Coccidioides* spp.	β(1→6)-glucan, gpA and gp120 (*P. carinii*), glucuronoxylomannan, and mannoprotein (*C. neoformans*)	Isolated from lung lavage fluids	(48–54)

MBL	*A. fumigatus, P. carinii, C. neoformans*, and *C. albicans*		Purified from plasma	(55–58)

DC-SIGN	*A. fumigatus, S. cerevisiae, C. neoformans, C.albicans, T. marneffei*, and *C. topicum*	Galactomannans (*A. fumigatus*), mannoprotein (*C. neoformans*), and N-linked mannans	DCs, AMs	(5, 39, 59–64)

Mincle	*A. fumigatus, Malassezia* spp., *F. pedrosoi, P. carinii*, and *C. albicans*	α-mannose, glyceroglycolipid and mannosyl fatty acids (*Malassezia* spp.), MSG/gpA (*P. carinii*)	Corneal epithelial cells, monocytes, macrophages, neutrophils, myeloid DCs, and some B-cell subsets	(36, 65–69)

MCL	*C. neoformans, C. albicans*	α-mannans	Plasmacytoid dendritic cells	(70, 71)

CR3	*A. fumigatus, M. furfur, S. cerevisiae, P. brasiliensis, H. capsulatum*, and *C. albicans*	pH-regulated Ag 1 (*C.albicans*)	Neutrophils, macrophages, natural killer cells, and monocytes	(5, 72–78)

Lox-1	*A. fumigatus*		Corneal epithelial cells	(79, 80)

Langerin	*M. furfur, S. cerevisia*, C. albicans, *C. glabrata, C. krusei, C. parapsilosis*, and *C. tropicalis*	β-glucan	Langerhans cells	(81)

MelLec	*A. fumigatus*	1,8-dihydroxynaphthalene-melanin	Endothelial cells, macrophages	(82)

**Figure 1:**
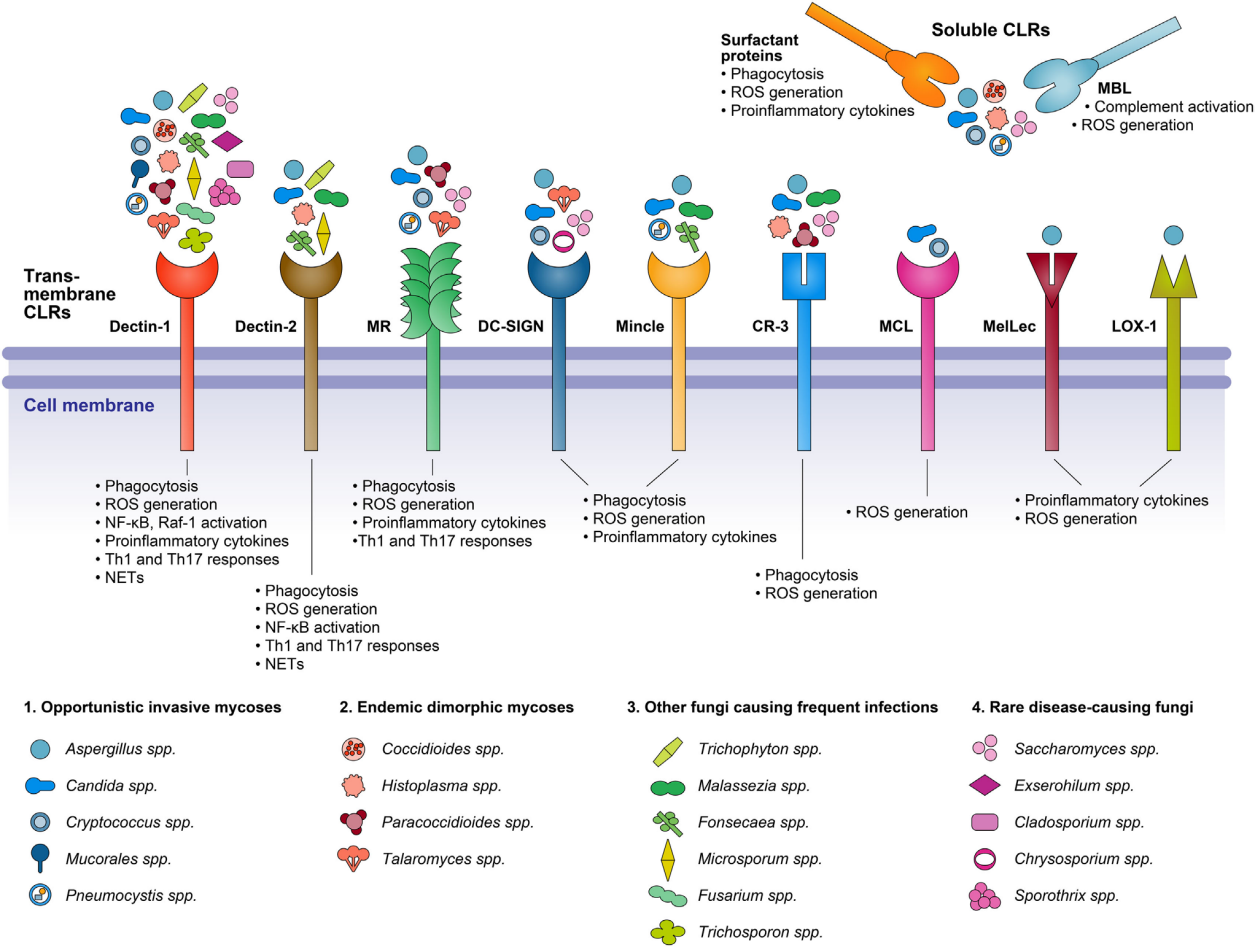
A diagrammatic representation of C-type lectin receptors (CLRs) involved in the recognition of various fungal species, and the resepective cellular responses triggered on receptor–ligand binding.

Fungal pathogens have a huge influence on human life, since they can infect the human body and cause various diseases from superficial infections to invasive and systemic infections. Infections of the skin and nails are the most common fungal diseases which affect ~25% of the general population worldwide (83). Invasive fungal infections have a lower incidence than superficial infections; however, they are of greater concern because they are associated with high morbidity and mortality. They are mostly caused by opportunistic fungal pathogens that take advantage of a debilitated immune system to proliferate in the human host and cause disease (84). Among the fungal species, only several 100 species are associated with human fungal diseases and just a minor number of species cause the most common invasive infections in immunocompromised individuals (85). The most notorious genera that are responsible for more than 90% of all reported fungal-related deaths are *Cryptococcus, Candida, Aspergillus*, and *Pneumocystis* (84). This increased prevalence of fungal infections has motivated the study of host–pathogen interactions in order to understand the protective and nonprotective mechanisms of antifungal immune responses in the human body. Investigation of the fungal recognition by the innate immune system led to the discovery of CLRs, the best-characterized PRRs for fungi. CLRs recognize carbohydrate polymers (mannan, glucans, and chitins) present in the fungal cell wall, resulting in the induction of innate and adaptive immunity to clear the pathogen (Figure 1; Table 1) (86).

In the following sections, we will summarize the current knowledge about the interaction of important human pathogenic fungi with CLRs. We further include information on CLR-associated single nucleotide polymorphisms (SNPs) and their effect on the susceptibility to fungal infections.

## Opportunistic Invasive Mycoses

### *Aspergillus* spp.

*Aspergillus* species (*Aspergillus* spp.) are ubiquitous molds commonly found in the soil. They produce a large number of conidia, which are released and dispersed into the air by wind leading to a deep penetration into the respiratory tract upon inhalation (87). These conidia are effectively cleared from the lungs of immunocompetent individuals. However, patients with a compromised immunity are at risk of developing an acute invasive aspergillosis (AIA). AIA is characterized by hyphal invasion of lung tissues and even dissemination to other organs (87). *Aspergillus fumigatus* (*A. fumigatus*) accounts for about 65% of all invasive infections in humans and is the most frequently encountered *Aspergillus* spp. in pulmonary infections. *A. flavus, A. niger, A. terreus*, and *A. nidulans* are less frequent causes of infections (87). The primary innate immune response is mediated mainly by macrophages, DCs, and neutrophils, taking place after *Aspergillus* spp. encounters these cells. Several of the *Aspergillus* cell wall components, such as β-glucans, chitins, and mannans act as ligands that are recognized by CLRs. Ligation results in the activation of cellular immune responses, such as phagocytosis, extracellular trap formation, conidial killing, and the production of proinflammatory and anti-inflammatory cytokines, such as TNF-α, IFN-α, IL-6, and IL-18 (88–91). Fungal recognition by specific CLRs can depend on morphological changes of *Aspergillus* spp., since different growth forms expose diverse PAMPs at variable amounts on their surface. For example, the surface of the *Aspergillus* dormant conidia does not present β-glucan, but is accessible for receptor recognition after the loss of hydrophobic cell wall components (outer layer of rodlets/hydrophobins and melanin) during the swelling of conidia and the development of germ tubes (89, 92, 93). Several CLRs are involved in the recognition of *Aspergillus* spp. such as the transmembrane receptors Dectin-1, Dectin-2, MR, DC-SIGN, and the soluble collectins MBL and the lung surfactant proteins (SP) SP-A and SP-D (94) (Table 1). The most studied *Aspergillus* receptor is Dectin-1. It is present on the surface of myeloid cells recognizing β-1,3-glucan, a common component of the cell wall of several fungi (15). However, another *Aspergillus* cell wall-associated polysaccharide, the galactosaminogalactan, has also been identified as a ligand and prevents host inflammatory responses *in vitro* and *in vivo*, in part by avoiding cell wall β-glucans recognition by Dectin-1 (95). Several observations suggest a significant role for Dectin-1 in protective immunity against *A. fumigatus* (96–98). *A. fumigatus* also induces the expression of cytokines (TNF-α and IL-12) and genes related to fungal recognition and phagocytosis in immature human DCs (99). The transcription of Dectin-1 in response to *A. fumigatus* likely occurs *via* granulocyte-macrophage colony stimulating factor (GM-CSF)/PU.1, where GM-CSF potentiates the expression of PU.1, which carries out transcription of Dectin-1 augmenting Dectin-1 protein expression and responsiveness in THP-1 cells (100, 101). In HEK293T cells, the activation of AP-1 by heat-killed swollen conidia was inhibited by treatment with Syk inhibitor, indicating that the Syk signaling pathway is required for AP-1 activation in a Dectin-1-dependent manner (102). Silencing of Dectin-1 in murine macrophages resulted in a reduced expression of proinflammatory cytokines, and an observed inhibition of phagocytosis (103). Likewise, *A. fumigatus* conidia and germ tubes stimulated NF-κB activation, mediated the secretion of proinflammatory cytokines involved in the recruitment of neutrophils, and led to ROS production by human monocyte-derived macrophages, murine macrophages, and alveolar macrophages (AMs) (89, 92, 93). Clinical studies showed that individuals who developed AIA during the course of chemotherapy often displayed a defective expression of Dectin-1. The frequency of Dectin-1-expressing monocytes was reduced in patients with AIA compared to controls (65.6 vs. 87.5%) (104). This important role of Dectin-1 was confirmed by transfecting murine AMs with a vector encoding full-length Dectin-1 (105). Results demonstrated that Dectin-1 overexpression enhanced the generation of proinflammatory cytokines TNF-α and IL-1β, and enhanced the killing ability of macrophages during *A. fumigatus* exposure (105). The epithelial lining of human airways is another important spot for host-pathogen interactions, and Dectin-1 is also expressed in lung tissues (12). One study found that *A. fumigatus* induces the expression of Dectin-1 *via* TLR-2 in human bronchial epithelial cells, resulting in the stimulation of proinflammatory responses and ROS generation in response to *A. fumigatus* indicating its important role in the innate immune response in non-phagocytic cells (106). Furthermore, some findings indicate that the pulmonary infection of mice with *A. fumigatus* induces concurrent Th1 and Th17 responses that depend on Dectin-1 (107). With regards to *Aspergillus*-induced fungal keratitis, recent findings demonstrated that Dectin-1 is expressed in the cornea of rat and mice, where it is involved in the detection of invading fungi (108–110). Also Dectin-2 triggers a response to *A. fumigatus* infection. Human plasmacytoid dendritic cells (pDCs) recognize *A. fumigatus* hyphae *via* Dectin-2, resulting in cytokine release and extracellular trap (pET) formation (88). The noticeable Dectin-2 expression of AMs in human lung during *A. fumigatus* invasion suggests a prominent contribution to antifungal defenses in pulmonary aspergillosis (90). Moreover, Dectin-2 ligation leads to NF-κB activation and ROS production in response to *A. fumigatus* infection in human macrophages (111). An *A. fumigatus*-specific ligand has not been described until now, but Dectin-2 binds to high-mannose structures distributed in several fungal species, including *Aspergillus* spp. (33). Collectins, such as SP-A, SP-D, and MBL also bind to *A. fumigatus* (55, 112). One study about the contribution of MBL in the antifungal defense in invasive pulmonary aspergillosis (IPA) showed that in murine models of IPA, rhMBL-treated (recombinant human MBL) mice showed 80% survival compared to untreated IPA mice. A clear increase of TNF-α and IL-1α in treated IPA mice and a significant decrease in pulmonary fungal hyphae and IL-10 could be observed (113). *In vitro*, there was an enhanced uptake of *A. fumigatus* conidia by polymorphonuclear neutrophil (PMNs) in the presence of rhMBL, indicating a protective role of this receptor during IPA, possibly through MBL-mediated lectin complement activation (113). SP-A and SP-D also enhanced agglutination and binding of conidia to AMs and neutrophils and increased the phagocytosis, oxidative burst, and killing of *A. fumigatus* conidia by human neutrophils and AMs (91). The SP-D-mediated protective mechanism is dependent on calcium-activated protein phosphatase calcineurin (114) and include enhanced phagocytosis by recruited macrophages and neutrophils and enhanced local production of the Th1 cytokines TNF-α and IFN-γ in the supernatant from mice lung cell suspension (115). Corneal epithelial cells also express SP-D and in the setting of fungal keratitis *A. fumigatus* may induce these cells to express inflammatory cytokines *via* the SP-D and NF-κB pathway (116, 117). Since β(1→6)-glucan is a ligand for SP-D and since many fungi, including *Aspergillus* spp., have this carbohydrate structure in their cell wall compositions, it is expected that SP-D recognizes all *Aspergillus* spp. (48).

Several other CLRs ligate *Aspergillus* spp., but for each only a few data are available. DC-SIGN, another transmembrane receptor of the CLR family expressed on the surface of DCs, contributes to the binding of *A. fumigatus* conidia in human DC (59). However, DC-SIGN is also expressed in AMs and lung tissue, suggesting a contribution of DC-SIGN in the initial stages and in fungal spreading during AIA (60). Galactomannans appear to be the main DC-SIGN ligand on the cell wall of *A. fumigatus* conidia (60). Additionally, CR3 influences adaptive responses to *Aspergillus*. Blocking of CR3 significantly reduced *Aspergillus*-induced Th1 and Th17 responses independently from complement activation, demonstrating that CR3 might play a significant role in the adaptive host defense against *A. fumigatus* (72).

In fungal keratitis models, LOX-1 was increased in *A. fumigatus* infected corneas of C57BL/6 mice and human corneal epithelial cells, indicating a possible role of this receptor in controlling the infection (79, 80). In addition, Mincle and MR may play a role in the early innate immune response of the corneal resistance, since their expression increased significantly during the initial period of *A. fumigatus* infection, along with an increased expression of TNF-α and IL-1β in human and rat cornea (38, 65). *A. fumigatus*-specific ligands for these receptors have not been described up to now.

Recently, the CLR Clec1a, also called melanin-sensing C-type lectin receptor (MelLec), has been decribed to play an important role in the detection of *A. fumigatus* through recognition of the naphthalene-diol unit of 1,8-dihydroxynaphthalene-melanin in conidial spores of *A. fumigatus*. MelLec is ubiquitously expressed by CD31+ endothelial cells in mice and is required for protection against disseminated infection with *A. fumigatus*. MelLec is also expressed by myeloid cells in humans and a SNP within the coding region of this receptor (rs 2306894) was identified that significantly increased the susceptibility of stem-cell transplant recipients to AIA (82). AIA is of great interest for immunogenetic studies due to its high prevalence. A moderately large number of studies have investigated the association of SNPs and other genetic variations of different CLRs in order to get some benefit for preventive strategies. For Dectin-1, the *CLEC7A* rs3901533 (T/T) and rs7309123 (G/G) genotypes and the presence of Y238X (rs16910526) polymorphism resulted in a significantly increased risk of AIA in a Caucasian population (118–120). Two SNPs of *CD209* encoding DC-SIGN (rs735239 and rs735240) are associated with a higher susceptibility to fungal keratitis in the northern Han Chinese population (121). Association analysis revealed that carriers the *CD209* rs4804800 (G), rs11465384 (T), rs7248637 (A), and rs7252229 (C) alleles and the variant *CD209*-139A/G (rs2287886) in the Caucasian population had a significantly increased risk of contracting IPA (118, 122).

Several studies show that distinct alleles, genotypes, and genotype arrangements of *SFTPA2* and *MBL2* may contribute to a susceptibility of the host to aspergillosis. A significant association of *SFTPA2* 1649G and *SFTPA2* 1660G and *MBL2* 1011A alleles with allergic bronchopulmonary aspergillosis patients suggests that defects in these innate immune molecules may lead to an increased genetic susceptibility to allergic airway inflammation and asthma (123–126). Another study implies that the presence of the T allele and CT genotype at position 868 of *MBL2*, the CC genotype at position 1649 of *SFTPA2*, and its combination with the CC or CT genotype on position 868 of MBL gene increases susceptibility specifically to chronic cavitary pulmonary aspergillosis in the Caucasian population (127). It was demonstrated that the presence of the codon 52 mutation (W/M52) within the MBL gene was particularly common in patients with chronic necrotizing pulmonary aspergillosis. Since the mutation results in changes in the protein structure, it is likely that a reduced amount of active protein is available for pathogen clearance (128).

Overall, Dectin-1 plays an important role in the local immune response during aspergillosis by inducing the expression of proinflammatory cytokines. Dectin-1 is the best-characterized CLR for the recognition of *A. fumigatus*, since it recognizes β-1,3-glucan, which is a major component of the inner cell wall of this fungus. Even a single polymorphism results in a significantly increased risk of contracting AIA, indicating the importance of this receptor in the contribution to antifungal defenses. With regards to the other CLRs recognizing *Aspergillus* spp., more studies are required in order to establish a concrete role of them during an AIA.

### *Candida* spp.

The most common species of *Candida* responsible for causing human diseases is *Candida albicans*. It is an opportunistic pathogen that commensally colonizes not only the skin but also the gastro-intestinal and urino-genital mucosal surfaces mostly in yeast form in healthy individuals. In cases of immunosuppression or weakening, the yeast forms can convert into virulent hyphae that can cause either muco-cutaneous infection or disseminate to internal organs causing candidaemia (129). In addition to phenotypic switching between yeast and hyphal forms, *C. albicans* virulence factors include adhesion properties, secreted lipases, and aspartyl proteases (130). Other clinically relevant *Candida* species include *C. krusei, C. glabrata, C. tropicalis, C. parapsilosis*, and others (131). Among the *Candida* species-recognizing CLRs are Dectin-1, Dectin-2, MCL, Mincle, MR, DC-SIGN, CR3, MBL, and Langerin.

Dectin-1 is a type II transmembrane receptor expressed on several antigen-presenting cells of myeloid origin, including macrophages, monocytes, neutrophils, mast cells, DCs, as well as pulmonary epithelium (12, 16). Like *A. fumigatus* and other fungi, Dectin-1 also recognizes *C. albicans* by binding to β-1,3-glucan (Table 1) (132).The binding of β-1,3-glucan to Dectin-1 is Ca^2+^-independent (133). Notably, Dectin-1 recognition of the yeast form of *C. albicans* induces responses such as phagocytosis and oxidative burst in mouse phagocytes, ultimately resulting in the clearance of the yeast cells; in contrast, filamentous forms may mask the β-glucans by mannans and affect certain β-glucan-mediated responses (17, 134, 135). However, *C. albicans* germ tubes can be resognized by Dectin-1 in Syk-dependent mechanism and initiate Th-17 response (136).

Dectin-1 recognition of *C. albicans* or its ligand β-1,3-glucan initiates several distinct immune responses. β-1,3-glucan-Dectin-1 binding leads to NFκB-mediated ROS production and proinflammatory cytokine release, such as IL-12, TNFα, and IL-6, *via* the Syk-CARD9 pathway in mouse DCs and macrophages, as well as in human intestinal cells. The response is enhanced in co-operation with TLR-2 (137–141). Interestingly, *C. albicans* activation of Dectin-1 can also result in anti-inflammatory responses, like IL-10 release by macrophages and peripheral blood mononuclear cells (PBMCs) or the production of IL-1 receptor antagonist (IL-1Ra) (142–144). Furthermore, ligation of Dectin-1 on APC by *C. albicans*, but also by other fungi and even the endogenous ligand galectin-9 drives T cell differentiation into a TH2/TH17 response (145–148). PKCδ is essential for CARD9-dependent NFκB activation (149). *C. albicans* also induces mast cell activation in rat and mice that leads to a differential cytokine production depending upon the fungal morphology, and induces phagocytosis and nitric oxide production in a TLR-2 and Dectin-1-dependent manner (150, 151). A similar co-operation of Dectin-1 with TLR-2 and TLR-4 can be observed in human mononuclear cells, PBMCs, and macrophages on stimulation with β-1,3-glucan (152, 153). The Dectin-1-Syk-CARD-9 pathway can also activate IRF5 to produce IFN-β (154) or ERK to generate proinflammatory responses against *C. albicans* (155). Moreover, Dectin-1 also mediates the β-1,3-glucan-medidated opsonization-independent phagocytosis by human neutrophils and retinal microglia (156, 157). The Syk-dependent pathway is also involved in β-1,3-glucan-containing phagosome maturation and recruitment of TLR-9 in RAW cells (158). Additionally, Dectin-1 involvement with *C. albicans* activates many other signaling pathways. Dectin-1 binding with *C. albicans* can activate NFAT transcription factors induce IL-2, IL-10, and IL-p70 release in collaboration with TLR-2 in mouse DCs (15, 159). β-1,3-glucan-induced human DCs activate NFκB *via* Syk as well as Raf-1 *in vitro*. In fact, Raf-1 activation represses Syk-induced RelB activity, although not completely, and increases p65 transactivation activity to induce IL-12p40 and IL-1β production (160). Several studies based on human and mouse cell lines have demonstrated that Dectin-1 is important in activating the inflammasomes such as the noncanonical caspase-8 inflammasome that promotes Th-17 responses which are essential for antifungal immunity (136, 161–166). Th17 responses are important against cutaneous infection, while Th1 responses are directed against systemic infection (134). Dectin-1 also co-operates with other CLRs such as SIGNR1 in mouse macrophages enhancing the oxidative burst against *C. albicans* (167). Some studies have also demonstrated a Dectin-1-dependent CR3 activation on mouse neutrophils and subsequent killing of *C. albicans* by these cells (168, 169). Human neutrophils release neutrophil extracellular traps in response to *C. albicans in vitro*, triggered by the ROS production on recognition of β-glucan by Dectin-1 and CR-3 (170, 171). Interestingly, Dectin-1 stimulation with *C. albicans* or β-1,3-glucan can also generate certain immunomodulatory responses, e.g., IL-10 production and reduction in ROS production *via* SHIP-1 activation in mouse GM-CSF-derived bone marrow cells (172). Additionally, *C. albicans*-Dectin-1 engagement induces human as well as mouse granulocytic myeloid-derived suppressor cells to dampen the pathogenic hyperinflammatory NK and Th17 responses (173). Moreover, a recent study has also demonstrated a role of Dectin-1 in adaptive immunity by controlling CD4+ T cell responses in the murine gut (174). All these results indicate how *C. albicans* can influence the immune responses by engaging the same receptor on different cell types.

Mouse knockout studies have shown contrasting results. Dectin-1 deficient mice display defective macrophage activation with impaired subsequent inflammatory responses and present enhanced fungal burden and dissemination after *C. albicans* infection (163, 175). However, further mouse studies implied that Dectin-1 deficiency probably plays a minor role in systemic *Candida* infection but may control the mucosal infections (176). The differences in the results may be attributed to different mouse and *C. albicans* strains used in experiments (177). Dectin-1-deficient mice also are more susceptible to *C. glabrata* infections and show impaired inflammatory responses (178).

Polymorphisms in Dectin-1 have been studied with respect to *Candida* infections. The first study to report an early-stop-codon mutation Y238X in a family with recurrent vulvovaginal candidiasis (RVVC) among four women demonstrated that the monocytes and neutrophils from homozygotes lack Dectin-1 expression and are defective in cytokine production such as IL-17 upon *C. albicans* stimulation *in vitro*. However, phagocytosis and killing of fungi is normal (179, 180). Another report demonstrated that heterozygotes for Y238X receiving hematopoietic stem cell transplantation display an increased incidence of gastrointestinal *Candida* colonization. The monocytes of homozygotes show less IL-1β production and lack of TLR-2-Dectin-1 synergism complementing the previous *in vivo* and *in vitro* studies describing the role of Dectin-1 in *Candida* infections (181). In an HIV-infected African population, a mutation I223S has been associated with a lower IFNγ response to *C. albicans* stimulation of whole blood and tends to provide protection against oropharyngeal candidiasis (182).

The hyphal form of *C. albicans* can be recognized by Dectin-2, which binds to high-mannose structures such as α-mannans in a cation-dependent manner and is expressed predominantly on macrophages and DCs (Table 1) (11, 33). Several studies in mouse DCs and macrophages have demonstrated that Dectin-2 mediates its signaling *via* the ITAM-bearing adapter FcRγ and the Syk-CARD9 pathway but the subsequent responses seem to differ depending upon the cell type, fungal morphology, and methodologies (11, 34, 183, 184). Nevertheless, *C. albicans* recognition by Dectin-2 can induce phagocytosis, proinflammatory cytokine production, such as IL-6, IL-23, TNFα, and IL-12 as well as protective Th-17 responses (34, 183). In addition, Dectin-2-deficient mice show decreased survival and high kidney fungal burden after 10 days of infection with *C. albicans* (34). Indeed, it was later shown in two independent studies that Dectin-2-deficient mice are also susceptible to *C. albicans* and *C. glabrata* systemic infections, showing high fungal burdens in kidneys and reduced neutrophilic phagocytosis (185, 186). Similar to Dectin-1, Dectin-2 can also induce type I IFN responses in mouse macrophages and DCs by activating IRF5 in response to *C. albicans* (154). PLCγ2 is essential for Dectin-2-mediated NF-κB, MAPK and ROS activation in mouse macrophages when infected with hyphal *C. albicans* (187). Zhu et al. showed that Dectin-2 and MCL (Dectin-3) heterodimers recognize *C. albicans* α-mannans more effectively than either receptor alone and that MCL-deficient mice are highly susceptible to systemic candidiasis (70). However, most of these studies have been performed in mouse models and provide a picture of Dectin-2 and MCL roles in murine *Candida* infections, and studies regarding their role in the human host and the impact of mutations in the human receptors will be needed in order to complete the picture.

Mannose receptor is a mannan-binding lectin found on phagocytic cell surfaces and recognizes *C. albicans* α-mannans (Table 1) (39). Early studies have demonstrated the involvement of MR in cytokine release, non-opsonic phagocytosis, and killing of *Candida* spp. by phagocytic cells (Figure 1) (40, 188, 189). Human DCs phagocytose and kill *Candida via* MR leading to subsequent responses such as Th1 immunity and ROS production (39, 190–192). Dectin-1 engagement with *C. albicans* on mouse macrophages induces surface MR shedding which could be the reason for downregulation of MR surface expression observed on rat macrophages after *C. albicans* ingestion (193, 194). Dectin-1 was later shown to be the main phagocytic receptor, while MR is recruited to phagosomes in mouse macrophages in later stages and mediates the secretion of immunomodulators such as TNFα and MCP-1 (195). Indeed, MR-deficient mouse macrophages are able to take up and phagocytose *C. albicans* normally (195, 196). However, in human phagocytic cells, MR induces Th17 responses upon stimulation with *C. albicans in vitro* by inducing IL-1β and prostaglandin E2 production, which is enhanced by Dectin-1/TLR-2 synergism (162, 197–199). Neumann et al. reported the formation of unique MR-induced pseudopodial protrusions called fungipods in human monocyte-derived DCs in response to *C. albicans* yeast, which may have role in fungal phagocytosis. This response is species-specific with *C. parapsilosis* showing stronger fungipod formation compared to *C. albicans* and *C. tropicalis* (41). In fact, the innate immune recognition of *C. parapsilosis* complex and *C. albicans* by human PBMCs differ with respect to the receptors involved and the induced cytokine production; for example, MR is important for TNFα and IL-1β production upon *C. parapsilosis* stimulation (200). Interestingly, IFNγ stimulation of human monocyte-derived DCs and macrophages increases the candidacidal activity of these cells by increasing non-opsonic phagocytosis and ROS production which is related to a reduced expression of MR (201, 202). So far, no genetic studies have been performed to understand the significance of MR polymorphisms in *Candida* infections.

*Candida albicans* yeast and hyphae are also recognized by the collectin MBL (56, 203). Several studies have demonstrated the binding of *Candida* spp. to MBL followed by activation of the complement system and subsequent opsonophagocytosis of fungi by phagocytic cells *in vitro* (204–206). Li et al. demonstrated MBL-dependent opsonophagocytosis of *C. albicans* by human neutrophils but without complement activation. This response was coupled with intracellular Dectin-1-dependent ROS production (207). Parenteral administration of MBL increased the resistance of mice in a model of disseminated candidiasis (56). In fact, mice deficient in MBL-A and MBL-C (mice homologs to human MBL) are more susceptible to systemic *Candida* infection (208). MBL is expressed in the mouse gut and its blocking or elimination leads to increased *C. albicans* colonization (209). MBL can also modulate the *C. albicans-*triggered TLR-generated proinflammatory signals by THP-1 cells (210). MBL concentrations are greatly affected by promoter polymorphisms in the *MBL2* gene and the resulting lower MBL levels are linked to the risk to develop several infectious diseases (128, 211, 212). Reduced levels of MBL were observed in the cervicovaginal lavage of RVVC patients, while the levels were higher in VVC patients compared to healthy controls (213–215). Moreover, MBL deficiency is also associated with the development of abdominal yeast infection in peritonitis patients (216). However, MBL serum levels and genotypes were not associated with intra-abdominal candidiasis in a Swiss cohort (217). The RVVC patients also have a higher frequency of *MBL2* mutations compared to both VVC and healthy groups (214). Furthermore, the *MBL2* codon 54 allele B is associated with a higher susceptibility to RVVC as observed in Belgian, Latvian, and Brazilian women (215, 218, 219). A recent meta-analysis of five different studies also concluded the correlation of allele B of codon 54 to be associated with both RVVC and VVC (220). Only a couple of studies have addressed the polymorphisms in components of MBL complement pathway in development of invasive fungal infections (221, 222). The polymorphisms in MBL complement pathway components have been associated with other infectious diseases as well, such as tuberculosis and leprosy (223, 224) and further studies exploring the effects of mutations in complement proteins on the pathogenesis of fungal infections are still needed.

Another CLR, DC-SIGN, can recognize the N-linked mannans in the *C. albicans* cell wall (39, 225). It mediates the internalization of conidia by human DCs, which are abundantly present in mucosal tissue (Table 1) (61), although human DCs exhibit less-efficient phagocytic activity compared to monocytes and macrophages (226). Gringhuis et al. showed that *C. albicans* stimulation can modulate TLR-dependent pathways by Raf-1 activation in human DCs. There is also evidence for possible anti-inflammatory effects upon DC-SIGN ligation. However, data so far are derived from non-fungal ligands (227). The fungus-induced IL-10 production is mediated through coactivation of DC-SIGN and TLR signaling pathways (228). The mouse homolog of DC-SIGN, SIGNR1 works in co-operation with Dectin-1 and TLR-2 in mouse macrophages to induce responses such as oxidative burst and TNFα production *in vitro* (167, 229). Knockout mouse studies and genetic studies may enlighten us more regarding the importance of this CLR in these fungal infections.

*Candida albicans* is also recognized by Mincle, another CLR expressed on several immune cells (Table 1), although the related ligand has not yet been discovered (230). An *in vivo* study demonstrated a non-redundant role of Mincle against *Candida* infection as Mincle-deficient mice were highly susceptible to systemic candidiasis. Mincle induces TNFα production in mouse macrophages upon *C. albicans* hyphae stimulation *in vitro* but is not important for phagocytosis, indicating that Mincle has a role in initial macrophage binding and early responses to the fungus (66). A similar effect was observed in human monocytes where the stimulation with *C. albicans* yeast leads to TNFα production but is related to poor yeast uptake. However, human neutrophils expressing Mincle show a fungicidal activity correlated with phagocytosis of the yeast (231). Taken together, more studies are needed to discover the *C. albicans* ligand(s) for Mincle and the related pathways as well as *in vitro* and *in vivo* immune responses and further work is necessary to elucidate the role of Mincle polymorphisms in fungal infections.

CR3 is an important CLR expressed on neutrophils, macrophages, NK cells, and monocytes and is involved in adhesion and phagocytosis of *C. albicans* (5, 73, 232). CR3 has a role in *C. albicans* hyphae recognition as the human lymphocyte adhesion of *C. albicans* hyphae was abrogated upon blocking CR3 with monoclonal antibodies (233, 234). Soloviev et al. showed that *C. albicans* releases a soluble CR3-binding mannoprotein called pH-regulated Ag 1 (Pra1), which mediates CR3-dependent adhesion and migration of THP-1 cells and neutrophils toward *C. albicans* (74, 235). Pra1 is expressed highly and exclusively on *C. albicans* hyphae (236). Soluble Pra1 was found to be beneficial for fungal survival as it inhibits the human neutrophil activation upon stimulation with Pra1 overexpressing *C. albicans* hyphae (235). Additionally, a CR3 knockout mouse study demonstrated that these mice show an increased susceptibility toward *C. albicans* systemic infection (168, 237) and their neutrophils display impaired adhesion, migration, and oxidative burst when challenged with *C. albicans in vitro* (237). Several studies have shown β-glucan to be another potential ligand for CR3 (238–240). Dectin-1 and CR3 co-localize on yeast *C. albicans* phagocytic cups in mouse peritoneal macrophages (195). Further studies support the interaction of Dectin-1 and CR3 in *C. albicans* infection as mentioned earlier. Dectin-1 activates CR3 for recognition of *C. albicans* yeast components and together they induce neutrophil cytotoxic responses in mice (168, 169).

Langerhans cells (LCs), found in epidermis and mucosal linings, express Langerin, which can bind to β-glucans and recognizes Candida spp., including *C. albicans, C. glabrata, C. parapsilosis, C. tropicalis*, and *C. krusei*, among others, *in vitro* (81, 241, 242). Some studies have shed light on the roles of LCs in *Candida* infections (243, 244), but specific studies regarding the role of Langerin are lacking.

Taken together, *Candida* is recognized by a number of CLRs, each of which is able to generate fungal-specific immune responses. While Dectin-2 and CR3 can recognize and respond to the fungal hyphae, Dectin-1, MR, and DC-SIGN mainly recognize the conidial forms. For Mincle, MCL and Langerin little is known and they need to be further investigated for their roles in *Candida* infections. Moreover, more studies need to focus on the genetic component of the effect of CLRs on *Candida* infections.

### *Cryptococcus* spp.

Cryptococcosis is a worldwide distributed and invasive fungal infection that is caused by species of the genus *Cryptococcus*. Nearly 100 species have been described within this genus so far, but *Cryptococcus neoformans* and *C. gattii* species are considered to be the only disease-causing fungi (245). Although cryptococcosis is predominantly a disease of immunocompromised patients (AIDS-defining illness), a recent outbreak showed the capacity of some lineages of the fungus to act as primary pathogens in healthy individuals (246). Within the lung, *Cryptococcus* spp. can cause pneumonia in immunosuppressed patients, and the latent infection can then disseminate to other tissues, most particularly the central nervous system (CNS), where this fungus causes an infection of the meninges accompanied by elevated intracranial pressure and without a rapid treatment it becomes fatal (246).

The interaction of *Cryptococcus* with CLRs is poorly understood. Initial data demonstrated that *C. neoformans* binds to soluble collectin MBL and the ingestion of the acapsular form is inhibited by both soluble mannan and β-1,3-glucan, showing that ingestion of acapsular *C. neoformans* takes place *via* mannose and β-glucan receptors in murine macrophages (247).

The role of Dectin-1 is still controversial, since no significant differences were observed in the clinical course and cytokine production between Dectin-1-deficient and control mice in a cryptococcosis model (248), but another study found that *C. neoformans* spores are phagocytosed by murine AMs *via* Dectin-1 (18). The role of Dectin-2 is also poorly understood. Some results show that it may not be required for the production of Th1 and Th17 responses, proinflammatory cytokines or for the clearance of *C. neoformans* in Dectin-2 knockout mice (249). Dectin-3 seems to have a role as it was demonstrated that human and murine pDCs have a direct Dectin-3-dependent anti-cryptococcal activity by inhibiting the growth of *C. neoformans via* ROS production (71). A *Cryptococcus*-specific ligand for this receptor has not been described yet.

Mannose receptor also has a role in *Cryptococcus* infection. After a pulmonary infection with *C. neoformans*, MR knockout mice died significantly earlier than wild-type mice and had higher lung fungal burdens (250). This receptor was required for the presentation of *C. neoformans* antigens to T lymphocytes by primary DCs, since blocking this receptor reduced both uptake of *C. neoformans* and lymphocyte proliferation (251). Some data suggest that mannoproteins, secreted by *C. neoformans*, might be the ligands for MR, as T cell stimulation is inhibited either by competitive blockade of MR in APCs or by removal of carbohydrate residues from mannoproteins. These results imply a capacity of mannoproteins to bind MR and to be processed by APCs to stimulate primary T cells (42). However, multiple receptors on DC could recognize this ligand, since DC-SIGN was also determined to have an affinity for mannoproteins. Further, MR and DC-SIGN both colocalize with mannoproteins, supporting a role for each in mannoprotein capture (62).

The pulmonary surfactant proteins, SP-A and SP-D bind to both encapsulated and acapsular *C. neoformans* (49, 252) and SP-D binds to the high-molecular weight polysaccharide glucuronoxylomannan and mannoproteins on the fungal cell wall (50). However, some data suggest that these receptors actually increase the susceptibility to *C. neoformans* infection. SP-A inhibits the IgG-dependent phagocytosis of *C. neoformans* by AMs and SP-A^−/−^ mice exhibit wild-type vulnerability to *C. neoformans*; SP-D^−/−^ mice are even protected during *C. neoformans* infection and display decreased fungal burden compared to wild-type mice. SP-D^−/−^ AMs also demonstrate an enhanced ability to kill *C. neoformans* cells (253, 254). Indeed, SP-D increases vulnerability to *C. neoformans* infection by stimulating *C. neoformans*-driven pulmonary IL-5 and eosinophil infiltration (255). SP-D may also play a role in protecting *C. neoformans* cells during the early stages of infection by opsonization. It was found that SP-D increases phagocytosis of hypocapsular *C. neoformans* by murine macrophages and enhances fungal survival allowing to gain access to specific intracellular compartments where it can grow (256). Another study reports that both, the presence of capsules and a wild-type cell wall design, prevent MBL binding to *C. neoformans* (257).

Last but not the least, complement activation by *Cryptococcus* spp. was demonstrated in the presence of MBL *in vitro* (57). A *Cryptococcus*-specific ligand for this receptor has not been described up to now.

Given that Dectin-1, Dectin-2, SP-A, and SP-D studies showed controversial results and their interactions with *Cryptococcus* are poorly understood, further studies are necessary.

### *Mucorales* spp.

Mucormycosis is the second most-common form of invasive mold infections. The disease is characterized by vessel thrombosis and tissue necrosis resulting from extensive angioinvasion and further dissemination (258). The members of the *Mucorales* order of Zygomycetes are among the leading causes of mucormycosis in immunocompromised individuals apart from more common fungal genera, such as *Candida* or *Aspergillus*, with mortality rates ranging from 50 to 100% (259). Among *Mucorales* spp., although rare, *Rhizopus oryzae* accounts for 70% of mucormycosis infections. Mainly phagocytotic cells play an important role in restricting the infection (260). The studies on mechanistic details of fungal recognition by CLRs and their role in pathogenesis are still lacking. One study reported that patients with mucormycosis showed reduced expression of Dectin-1 on monocytes compared to healthy controls (104). However, further investigations into the role of C-type lectins and their polymorphisms in this infection are needed.

### *Pneumocystis* spp.

The genus *Pneumocystis* includes a variety of ubiquitous fungi that colonize and infect several mammalian host species. The species *P. jirovecii* particularly infects humans, whereas *Pneumocystis carinii* (*P. carinii*) and *P. murina* are associated with rats and mice, respectively. In the immunocompromised host, *Pneumocystis* pneumonia (PCP) is fatal if untreated. However, infection of an immunocompetent host can result in a self-limited mild or subclinical lower respiratory tract infection (261).

The first studies demonstrated an interaction of *Pneumocystis* cell wall isolates with macrophage β-glucan receptors, which induced a potent stimulation of TNF-α release in rat AMs in response to *P. carinii* (262, 263). During *P. carinii* infection, the expression of Dectin-1 is upregulated in macrophages of immunocompetent rat models (264). According to some studies, Dectin-1 is required for the protection against *P. carinii* infection, since Dectin-1-knockout mice are more sensitive to infection than infected wild-type mice, and production of ROS is completely abolished in Dectin-1-knockout macrophages incubated with *P. carinii* (176). Probably, the expression levels of Dectin-1 in AMs are under the control of the transcription factor PU.1 during a PCP infection, where the GM-CSF appears to play a major role in the regulation of PU.1 expression (265). Phagocytosis of *P. carinii* and generation of hydrogen peroxide by murine AMs is mediated by Dectin-1, since the blockage of Dectin-1 inhibits the binding and killing of *P. carinii* (266). The binding of Dectin-1 to *Pneumocystis* was tested by creating recombinant Dectin-Fc fusion proteins which bind *P. carinii* and enhance murine macrophage-dependent killing. These findings demonstrate that Dectin-1 binds β-glucan from *Pneumocystis*, enhancing host recognition and clearance of *P. carinii* (19). *P. carinii* β-glucan cell wall component challenge of rat alveolar epithelial cells resulted in a prominent nuclear translocation of p65 NF-κB with a subsequent increase in MIP-2 and TNF-α mRNA production. However, rat alveolar epithelial cells do not require Dectin-1 for MIP-2 production, which rather involves the participation of the alternative lactosylceramide β-glucan receptor (267, 268).

*Pneumocystis carinii* also enhances soluble MR production in human and murine macrophages (269). In human AMs, phagocytosis of *Pneumocystis* is mediated through MR and depends on Cdc42 and especially RhoB activation (270). *Pneumocystis* also stimulates NF-κB nuclear translocation in human AMs, which is mediated primarily through MR (43). A recombinant soluble MR-Fc fusion protein binds *P. carinii* and leads to an increased uptake by hPMNs (271). The role of MR was confirmed by the fact that binding and uptake of cultured *P. carinii* by human and rat AMs is reduced 90% by using competitive inhibitors of MR, emphasizing the role of the AMs in the first-line host defense (272). Other studies suggest that a reduced AM MR-mediated binding of *P. carinii* may contribute to the susceptibility of HIV-infected individuals to this pathogen (273). However, it was demonstrated that IL-8 release by human AMs following the stimulation with *Pneumocystis* requires the co-expression of MR and TLR-2, since the IL-8 release is reduced significantly upon blocking of TLR-2 and silencing of MR gene (274). These results support the idea that MR on human AMs may suppress the production of proinflammatory cytokines and may serve to regulate the innate inflammatory responses to *Pneumocystis* infection in the lungs (275). A *Pneumocystis*-specific ligand for MR has not been described up to now.

Some results indicate that SP-A and SP-D can modulate the virulence of *P. murina* and *P. carinii* during development of infection in SP-D- and SP-A-deficient and immunosuppressed mice. They attenuate the production of proinflammatory cytokines and ROS and RNS, indicating that both receptors are local effector molecules in the lung host defense against *Pneumocystis in vivo* (276–280). These results are supported by the fact that SP-A and SP-D can bind *P. carinii*, acting as opsonins and enhancing their phagocytosis by AMs (281–284). However, some data suggest that the increased SP-A and SP-D mediated aggregation of *P. carinii* fungal particles interferes with AM recognition and thus the SPs may contribute to the pathogenesis of *P. carinii* pneumonia (285–287). This view is supported by the fact that SP-A in immunosuppressed mice acts as a therapeutic agent in the beginning of *Pneumocystis* infection, but not in the middle or late stages of the infection (288). SP-D strongly interacts with gpA, the main glycoprotein antigen on the surface of *P. carinii*. The interaction of SP-D with *P. carinii* gpA is mediated by the carbohydrate recognition domain (CRD) of this collectin (51, 289). Similarly, the CRD of SP-A mediates binding to the main surface glycoprotein gp120 of *P. carinii* (52, 290).

Binding of MBL to *P. carinii* is followed by the activation of the respiratory burst, indicating that the MBL in serum has opsonizing properties and might contribute in controlling fungal spread from the lungs (58). A *Pneumocystis*-specific ligand has not been described up to now for this receptor.

Mincle binds whole *P. carinii* and a surface glycoprotein called MSG/gpA, a *Pneumocystis* cell wall component, which is expressed at enhanced levels during infection (67). Moreover, Mincle^−/−^ mice exhibit significantly higher *P. murina* burdens with elevated levels of TNF-α, IL-6, and IL-1Ra during infection, indicating that Mincle functions as an important signaling receptor in host defense against *Pneumocystis* infection (67).

Little is known about polymorphisms affecting *Pneumocystis* recognition, however, one study analyzed 53 HIV patients having CD4 counts <200 μL, in order to find a correlation between MBL and PCP. Of these 53 patients, 30 had PCP at admission, and 23 did not. Genotypes related with a low production of MBL were significantly more common in the PCP group than in the non-PCP group. Serum MBL levels were significantly higher in the non-PCP group. Genetic variations influencing MBL production also affect the susceptibility to PCP in HIV-advanced infection patients, and may be considered as a risk factor for PCP (291).

Overall, CLRs seem to be of importance for orchestrating the *Pneumocystis*-induced immune response. However, *Pneumocystis* cannot easily be propagated in culture, which has delayed the understanding of its pathobiology. Efforts to study *Pneumocystis* have been greatly limited by the inability to maintain *ex vivo* culture of the organism. Early attempts to isolate and propagate *P. jirovecii*, have been moderately successful, however, none of these models garnered sufficient recognition to become a standard method for the isolation of *Pneumocystis* (292). Nonetheless, studies of organisms isolated directly from the infected lung of patients or immunosuppressed research animals still allow for some insight into the pathobiology of *Pneumocystis* (293).

## Endemic Dimorphic Mycoses

### *Coccidioides* spp.

There are two species of *Coccidioides* (*C. immitis* and *Coccidioides posadasii*) that cause human disease. They have similar phenotypes and pathogenicities, but differ in genotype and geographic distribution. They are the etiologic agents of coccidioidomycosis, which ranges from asymptomatic infections to pneumonia and severe disseminated disease. These organisms are found in the soil, especially in low-moisture environments, so preventing exposure can be difficult due to the ubiquitous risk of dust inhalation by individuals living in endemic areas. The pathogenesis of coccidioidomycosis is complex and can be asymptomatic but also cause extrapulmonary dissemination (294).

The reasons of the complexity of the pathogenesis of coccidioidomycosis are not well understood; however, some data suggest the main involvement of Dectin-1. Some results suggest that an alternative splicing of the Dectin-1 gene enhances the susceptibility of C57BL/6 mice to coccidioidomycosis, regulating the cytokine responses of macrophages and mDCs to spherules, the pathognomonic structure of this fungus (295). RAW 264.7 macrophages overexpressing Dectin-1 produced more TNF-α than control macrophages in respond to *C. posadasii* spherules. Also, macrophages overexpressing Dectin-1 and activated with purified β-glucan from *C. posadasii* spherules produced a significantly higher level of TNF-α than control macrophages, indicating a role of β-glucan from *C. posadasii* as a ligand for Dectin-1 (20). Moreover, Dectin-1 activation is essential to leading the adaptive immune response toward Th1 and Th17 pathways, thus leading to the resolution of infections in mice (35, 296).

Other results suggest that there is an association between low serum MBL levels and symptomatic coccidioidomycosis, but in order to understand the role of MBL in the pathogenesis of this fungal disease, further studies are necessary (297). SP-A and SP-D also bind coccidioidal antigens (53). Deficiencies of MR and Dectin-2, either alone or in combination, affect cellular responses to formalin-killed spherules *in vitro* but do not make C57BL/6 mice more vulnerable to pulmonary coccidioidomycosis (298). A *Coccidioides*-specific ligand for these receptors has not been described up to now.

In conclusion, further studies are necessary to elucidate interactions of *Coccidioides* with CLTRs, since few receptors and no ligands have been studied. However, Dectin-1 seems to have an important role in this infection.

### *Histoplasma* spp.

Fungi of the genus *Histoplasma* cause histoplasmosis and are found throughout the world, but are most common in North America and Central America. *Histoplasma capsulatum* is a member of this group of fungal pathogens that cause respiratory and disseminated disease in mammals. It grows as a saprobic conidia-producing mycelium in the environment, and when the aerosolized mycelium fragments and conidia are inhaled, they reach the lower respiratory tract causing disease even in immunocompetent hosts (299).

Little is known about the receptors recognizing *Histoplasma* and its signaling response. However some CLRs, such as Dectin-1, Dectin-2, and some collectins are involved in *Histoplasma* immunity (Table 1). Dectin-1 and Dectin-2 exert several contributions to the development of antifungal Th1 and Th17 cells and vaccine resistance in mice against *H. capsulatum* (35, 300). CR3 and Dectin-1 act together to induce murine macrophages to TNF and IL-6 responses through a Syk-JNK-AP-1-dependent mechanism (75). Some data show that CR3 participates in phagocytosis and cytokine responses, but Dectin-1 takes part in cytokine production only on murine macrophage (21). *Histoplasma* pathogenic yeast cells secrete Eng1, a β-glucanase that hydrolyzes β-(1,3)-glycosyl linkages, which reduces levels of surface-exposed β-glucans on yeast cells, thereby enabling *Histoplasma* yeasts to escape detection by Dectin-1. *Histoplasma* yeasts deficient for Eng1 show an enhanced binding to Dectin-1 and an increased TNF-α and IL-6 production in murine macrophages and DCs (301, 302). Also, SP-A and SP-D demonstrate potent antifungal properties, since they cause a dose-dependent decrement in yeast viability, which is associated with an increase in the permeability of the yeast cells. Mice lacking SP-A manifest a modestly higher fungal burden in lungs than wild-type littermates (54). A *Histoplasma*-specific ligand for these receptors has not been described up to now.

Together, these studies indicate minor roles for CLRs in the control of *Histoplasma* infections, but further studies are needed to understand the significance of CLRs in *Histoplasma* infections.

### *Paracoccidioides* spp.

*Paracoccidioides* spp. is the causal agent of paracoccidioidomycosis (PCM), a systemic mycosis endemic to Latin America. It comprises two species: *Paracoccidioides brasiliensis* and the recently described *P. lutzii* (303). Manifestations of PCM include subclinical or asymptomatic infection. The symptomatic disease causes an acute/subacute or a chronic form, the latter involving the lungs as well as other organs. PCM is acquired after inhalation of infectious propagules in the environment, leading to a primary pulmonary infection (303).

Few studies have investigated the role of CLRs on *Paracoccidioides* infection. Human monocytes display a decrease in Dectin-1 expression as soon as 30 min after stimulation with *P. brasiliensis* (22). There is a trend toward an increased Dectin-1 mRNA expression in response to *P. brasiliensis* and this receptor is able to induce a balanced production of TNF-α, IFN-γ, IL-12, and IL-10 in human neutrophils and monocytes (22, 304, 305). By binding to Dectin-1, *P. brasiliensis* induces neutrophil extracellular trap (NET) release that is responsible for trapping yeast cells, promoting their immobilization, as well as contributing to their extracellular killing (306). Moreover, the fungal infection of Dectin-1^−/−^ mice results in enhanced tissue pathology and mortality rates. The deficiency of Dectin-1 has also reduced the production of Th1, Th2, and Th17 cytokines and the activation and migration of T cells to the site of infection (307). Altogether, these results suggest the participation of Dectin-1 in *P. brasiliensis* recognition, internalization, and consequent activation of the immune response against the fungus. A *Paracoccidioides*-specific ligand for Dectin-1 has not been described up to now, however, it is known that Dectin-1 binds glucan structures that are distributed in a wide range of fungal species.

The gp43 glycoprotein is the main antigenic component secreted by *P. brasiliensis*. gp43 binds to TLR2, TLR4, and MR receptors and all three receptors influenced a high production of IL-10 and TNF-α in human monocytes (44). The specific blockade of MR and CR3 impaired fungal recognition and modified the production of cytokines (308). The CR3 receptor may participate in phagocytosis of *P. brasiliensis* conidia through both opsonic and non-opsonic mechanisms, since treatment of murine macrophages with anti-CR3 and α-methyl-d-mannoside, a competetive inhibitor of the binding of mannose, decreased phagocytosis of *P. brasiliensis* (76). In the same way, the mannose-binding lectin complement pathway was demonstrated to play a key role in complement activation by *P. brasiliensis* (309). A *Paracoccidioides*-specific ligand for this receptor has not been described until now.

Overall, interactions between host immune cells and *Paracoccidioides* spp. are mediated by the recognition of Dectin-1 which controls internalization by phagocytes as well as lymphocyte proliferation during *P. brasiliensis* infection. However, further studies are necessary, since few receptors and no ligands have been studied.

### *Penicillium* spp.

*Penicillium* species are rarely considered as human pathogens except *Talaromyces (Penicillium) marneffei*, which can cause opportunistic infections, called penicilliosis, in immunocompromised patients, especially in HIV positive persons, but also in old and new born (310). The infection is most prevalent in South-east Asia and is characterized by symptoms, such as weight loss and fever, skin lesions, generalized lymphadenopathy and hepatomegaly, and respiratory signs such as hemoptysis (310, 311). The main virulence factor of *T. marneffei* is its temperature-dependent dimorphic growth, owing to which it grows as mycelium at 25°C while at 37°C it grows as yeast (311). *In vitro* experiments have shown that *T. marneffei* can be recognized by Dectin-1, DC-SIGN, and MR (Figure 1) (23, 45, 63). Koguchi and colleagues demonstrated that blocking MR with antagonists reduces the osteopontin production from PBMCs upon *T. marneffei* stimulation and suggested a mannoprotein as the possible ligand (45). Indeed, MR was later found to be involved in adhesion and phagocytosis of the fungus by human monocyte-derived DCs, while DC-SIGN only mediated adhesion (63). IL-12p40 production by bone marrow-derived dendritic cells (BMDCs) upon stimulation with *T. marneffei* is abrogated in Dectin-1 knockout mice (23). Together, these studies suggest that CLRs modulate the immune response to *T. marneffei*; however, further studies are needed to dissect the *in vivo* role of CLRs and their polymorphisms in *T. marneffei* infections.

## Other Fungi Causing Frequent Infections

### *Trichophyton* spp.

Dermatophytosis is one of the most common mycoses worldwide. Compromising keratinized tissues and characterized by establishing chronic inflammatory processes, it is highly resistant to standard antifungal therapies. The main etiological agent in humans is *Trichophyton rubrum*. It establishes infection after being inoculated in the host tissue, where it survives through the degradation of dead cells, consuming keratin and other host components (312).

Only few studies analyzed the roles of CLRs in *Trichophyton* spp. infections. Dectin-1 is involved in mediating inflammation induced by trichophytin, a *T. mentagrophytes* antigen with β-glucans and zymosan as the main components (24). *T. rubrum* hyphae are recognized by Dectin-1 and Dectin-2 in murine DCs, triggering production of inflammatory cytokines, mainly IL-1β and TNF-α. This inflammatory process is able to promote the clearance of the pathogen *in vivo* without the involvement of lymphocytes. Even though IL-17 is induced, it is not essential for infection resolution (313). Moreover, trichophytin enhances the Dectin-1 expression in mice, and the blockage of Dectin-1 inhibits the increased IFN-γ production in cervical lymph node cells from mice *in vitro* (314). Dectin-2 preferentially binds to hyphae of various fungal species, including *T. rubrum* (11).

### *Malassezia* spp.

The fungal genus *Malassezia* comprises yeast species that are part of the normal skin microbiota. However, *Malassezia* spp. can be involved in skin disorders, such as pityriasis versicolor, seborrheic dermatitis, atopic eczema, and folliculitis (315). *Malassezia* spp. may also cause invasive infections in infants and in immunocompromised individuals. The clinical spectrum ranges from asymptomatic infections to life-threatening sepsis and disseminated diseases (316).

Various *Malassezia* spp. (*M. japonica, M. slooffiae, M. furfur*, and *M. sympodialis*) induce a Dectin-1-dependent NLRP3 inflammasome activation with a subsequent IL-1β secretion in human APCs. This activation is dependent on Dectin-1, since the blocking of Dectin-1 decreased the IL-1β secretion upon *M. furfur* exposure (25). A *Malassezia*-specific ligand has not been described until now, but β-1,3-glucan is most likely present on their surface.

Several *Malassezia* spp. (*M. pachydermatis* and *M. furfur*) are recognized by Mincle and Dectin-2 through different ligands. A glyceroglycolipid and mannosyl fatty acids linked to mannitol are two Mincle ligands, and an O-linked mannobiose-rich glycoprotein is a ligand for Dectin-2. Both receptors cooperatively contribute to the TNF and IL-10 production in BMDCs from mice in response to *Malassezia spp* (36). Other results indicate that Mincle also recognizes *Malassezia* spp. (*M. pachydermatis, M. dermatis, M. japonica, M. nana, M. slooffiae, M. sympodialis, M. furfur*, and *M. pachydermatis*) through α-mannose but not mannan. Mincle may recognize a particular distribution of α-mannosyl residues on *Malassezia* spp. and use this to discriminate them from other fungi, inducing inflammatory responses (TNFα and IL-10) in murine macrophages (317).

Langerin plays a role in pathogen recognition by facilitating pathogen uptake and processing for antigen presentation (318). Langerin on primary LCs isolated from human epidermis interact strongly with *M. furfur via* β-glucan structures and the interaction can lead to the phagocytosis of the fungus (81). *M. furfur* is also recognized through MR and CR3 on THP-1 cells (77).

### *Fonsecaea* spp.

*Fonsecaea* spp. are found in soil and plants. Although they are considered a worldwide-distributed fungus, they are frequently found in tropical regions (319). *Fonsecaea pedrosoi* is a frequent causative agent of chromoblastomycosis (or chromomycosis), a chronic fungal disease limited to the skin and subcutaneous tissues. Initial lesions are habitually erythematous papules, which progressively enlarge to morphologies, such as verrucous nodules, cauliflower-like tumors, and psoriasis-like plaques (319).

Dectin-1, Dentin-2, and Mincle have a role in the recognition of this fungus (Table 1). Murine macrophages stimulated by co-culturing with muriform cells (the parasitic form of *F. pedrosoi*) show an elevated expression of the Dectin-1, and by blocking Dectin-1, the phagocytosis of muriform cells, was impaired, demonstrating that muriform cells are recognized by Dectin-1 *in vitro* (26). *F. pedrosoi* spores trigger Dectin-1 and Dectin-2 signaling and induce IL-6 production, but only the Dectin-2 signaling pathway promotes the differentiation of Th17 cells, indicating that the adaptive immune response to *F. pedrosoi* spores in this murine infection model is determined by Dectin-2 (37). A *Fonsecaea*-specific ligand has not been described until today.

Mincle acts as a major receptor involved in the innate immune response to *F. pedrosoi* through the Syk/CARD9 pathway in murine BMDCs (68). However, another study identified Mincle as a suppressor of antifungal defenses by suppressing IL-12. The absence of IL-12 leads to impaired Th1 responses. Dectin-1 binding of *F. monophora* activates the transcription factor IRF1, which is crucial for the *IL12A* transcription. However, simultaneous binding of *F. monophora* to Mincle induces a Mdm2 (E3 ubiquitin ligase)-dependent degradation pathway *via* Syk-CARD9-mediated PKB signaling, that leads to the loss of nuclear IRF1 activity, therefore, blocking *IL12A* transcription (320). A *Fonsecaea*-specific ligand has not been described up to now for this receptor.

Regarding Mincle, it is difficult to ascertain a particular role for this CLR in *Fonsecaea* infection, since it is not clear if this receptor is mainly involved in the recognition and subsequent clearance of this fungus or acts as a suppressor of antifungal defenses and is exploited for immuno evasive strategies.

### *Microsporum* spp.

*Microsporum* causes skin infections or dermatophytosis characterized by severe scalp itching and patchy scaly scalp skin which is highly contagious. The pathogenic species include mainly *M. cani, M. gypseum*, and *M. hominis* (321). The fungi secrete a number of enzymes and immunomodulators such as keratinolytic subtilase and keratinolytic metalloprotease as well as other cell wall glyco-proteins, endoproteases, and exoproteases (322). *M. cani* activates the NLRP3 inflammasomes in THP-1 cells. The production of IL-1β and its precursor are decreased in Dectin-1, Syk, and CARD-9 knockdown cells (323). Soluble Dectin-2 can bind the filamentous *M. audouinii* (11). Further research on CLR ligands, their recognition, and corresponding immune response in *Microsporum* infection are lacking.

### *Fusarium* spp.

*Fusarium* species can cause superficial, locally invasive infections in immunocompetent individuals, or disseminated infections in immunocompromised patients. The infection is called fusariosis and is characterized by keratitis, onychomycosis, fungimia with or without organ involvement, and other symptoms depending upon the fungal species, port of entry, and host immune status. In humans, *Fusarium solani* and *F. oxysporum* are responsible for most cases of infections by these species (324). *Fusarium* spp. secrete various mycotoxins as well as certain proteases and collagenases, which modulate the immune response and destroy tissue (324).

Dectin-1 expression is highly elevated in the corneal tissue from patients infected with *F. solani* when compared to healthy non-infected individuals (325). Human corneal epithelial cells secrete defensive antimicrobial peptides in response to heat-killed *F. solani* or zymosan and this effect was Dectin-1- and TLR-2-dependent (326). Another more recent study addressed the Dectin-1-dependent CXCL-8 release from the human bronchial epithelial cell line BEAS-2B in response to *F. proliferatum* and showed that the chemokine release is decreased to various degrees by inhibiting Dectin-1, Syk, MAPKs, PI3K, and NFκB, respectively (27). The expression of SP-D increased in rat corneal cells after *F. solani* infection but its further role in murine models as well in humans is still to be established (327).

### *Trichosporon* spp.

*Trichosporon* are ubiquitous dimorphic fungi, which also exist as commenals on the skin and in the gastrointestinal tract in humans. They induce superficial infections such as white piedra characterized by the presence of irregular nodules on the affected hair, as well as invasive infections such as allergic pneumonitis and trichosporonosis (invasive mycoses) especially in immuno-compromised patients and those with hematological malignancies (328, 329). *T*. *asahii, T. asteroids*, and *T. mucoides* are the major causes of trichosporonosis and opportunistic infections (330). Only a little is known about the interaction of these fungi with CLRs. Dectin-1 binds *T. asahii via* β-glucan recognition (28). Dectin-1-deficient mice with *T. asahii* induced hypersensitivity pneumonitis show decreased Th-17 cell populations and less monocytes/MDMs compared to wild-type mice (28).

## Rare Disease Causing Fungi

### *Saccharomyces* spp.

Classically, *Saccharomyces* spp. are considered safe, non-pathogenic organisms. Within this genus, *Saccharomyces cerevisiae* is the most important species (331). However, due to its ubiquity and long association with humans, *S. cerevisiae* has been implicated as a causative agent of infections in immunocompromised individuals, those with underlying diseases or medical conditions (332). Several cases of life-threatening invasive infections with *S. cerevisiae* resulting in pneumonia, liver abscess, and sepsis have been reported (333).

*Saccharomyces cerevisiae* cells cause a subtle upregulation of Dectin-1 from the moment of initial recognition in human DCs (334). Most studies have been performed by evaluating pure soluble and particulate β-glucans such as β-1,6-branched and β-1,3-d-glucan found in the *S. cerevisiae* cell wall (335), which can be directly recognized by Dectin-1 (29, 336). β-glucan induces Dectin-1 signaling pathways for the activation of TNFα in both human and mouse macrophages. The signaling pathways involve RTKs, ROS production, and NF-κB activation (337, 338). The Dectin-1-dependent response is essential for immunomodulatory effects on DC activation and macrophage phagocytosis. It induces the expression of immuno-regulatory cytokines, such as IL-10, TGF-β1, and IL-2 and can promote both Treg and Th17 responses (339–341). Moreover, the Dectin-1 response was investigated by observing the direct phagocytosis of β-glucan-coated particles by RAW macrophages expressing a GFP-Dectin-1 fusion protein. As expected, the β-1,3-beads induced a higher TNF-α response and a GFP-Dectin-1 recruitment to the phagosome, indicating that Dectin-1 recruitment is specific to β-1,3-glucan (342). In general, binding of particulate β-glucans to Dectin-1 triggers phagocytosis (343, 344). However, phagocytosis of β-glucan-bearing particles by human neutrophils is CR3-dependent, with a very minor role for Dectin-1, if any (78). Like *C. albicans*, also *S. cerevisiae* components in form of Zymosan are able to induce anti-inflammatory responses such as IL-10 release in human and mouse DCs and macrophages (345, 346).

Also, Langerin and DC-SIGN interact strongly with *S. cerevisiae* (64, 81). SP-D but not SP-A binds *S. cerevisiae*, and β(1→6)-glucan is a ligand for SP-D (48). Moreover, phagocytosis of unopsonized heat-killed yeast by murine macrophages is also mediated by MR (46).

Several studies have concluded that genetically determined low MBL concentrations in patients could be, at least in part, responsible for the enhanced immune reactivity to *S. cerevisiae* antigens (347–349). The analysis of *MBL2* polymorphisms revealed an association between three variants rs930508, rs1800450, and rs5030737, with a reduction in MBL serum levels in Crohn’s disease patients (350). However, these results are in contrast with other reports in which such an association was not found. Therefore, the relationship between enhanced immune reactivity to *S. cerevisiae* antigens and MBL is still controversial (351, 352).

Despite its low pathogenicity, *S. cerevisiae* constitutes one of the better studied microorganisms, since it was developed as a model organism for several traits. Zymosan is mostly prepared from *S. cerevisiae* cell walls and consists of a glucan with repeating glucose units linked by β-1,3-glycosidic linkages, which have served as a model for recognition of microbes by the innate immune system for over 50 years (353). Many studies have been conducted testing and evaluating the zymosan interaction with human receptors and Dectin-1 emerged as the most important receptor for detecting *Saccharomyces* spp. However, for the other CLRs more studies are required in order to stablish a concrete role for them.

### *Exserohilum* spp.

*Exserohilum* species are environmental fungi and, although rare, can lead to a number of human diseases such as skin and corneal infection, invasive disease, as well as allergic fungal sinusitis especially during impaired immunity, trauma, and atopy (354). Members of this genus are among the causes of phaeohyphomycosis which is characterized by the presence of dark septate mycelial elements in tissues (355). Not much work has been done to elucidate the mechanism of infection and immune responses against these fungi. A very recent work demonstrates the role of Dectin-1 in the recognition of *Exserohilum rostratum* (30). Mouse macrophages generate a Dectin-1 dependent TNF-α, IL-1β, MIP-1, and MIP-2 secretion in response to *E. rostratum* hyphae *in vitro* and the response is diminished in Dectin-1-deficient macrophages. However, wild-type and Dectin-1-deficient mice show no difference with respect to the type of inflammatory response and fungal control (30).

### *Cladosporium* spp.

The *Cladosporium* genus consists of ubiquitous fungi that are mainly plant pathogens but few species such as those belonging to *Cladosporium cladosporioides* and *C. herbarum* complexes may cause infections in humans (356). The clinical manifestations range from keratitis, opportunistic phaeohyphomycosis including superficial or deep infections such as those of the CNS, to acnes (356–359). *Cladosporium* conidia are widely present in the air and have also been associated with respiratory allergy (360). The *C. cladosporioides* cell wall is rich in β-glucans but unavailable for recognition on live spores. A mouse *in vivo* study demonstrated that *C. cladosporioides* induces airway hyperresponsiveness and eosinophilia in a Dectin-1-independent manner (361). Furthermore, the heat-induced availability of surface β-glucans is important for a Dectin-1-dependent pulmonary IL-17 response and Dectin-1^−/−^ mouse DCs show a decreased IL-17 response upon stimulation with heat-killed *C. cladosporioides* (31). The ligands on live *Cladosporium* cell surfaces and the corresponding CLRs, as well the subsequent immune responses are still to be investigated.

### *Chrysosporium* spp.

The members of this genus are saprophytic soil fungi and many species are keratinolytic (362). Several case reports of superficial infections affecting nails and skin as well as opportunistic infections in immunocompromised patients have been reported (362–365). Superficial infections are mainly caused by species, such as *C. keratinophilum, C. tropicum*, and *C. queenlandicum* (362). Invasive infections are rare but have been reported (366–368). A single study demonstrated that DC-SIGN recognizes *C. topicum* conidia probably by recognition of fungal cell wall mannans *in vitro* (59).

### *Sporothrix* spp.

These fungi are usually found living as a saprophytes thriving on decaying vegetation or soil. Sporotrichosis, caused by dimorphic *Sporothrix* spp., is one of the most prevalent forms of subcutaneous mycoses with a worldwide distribution particularly in tropical and subtropical regions (369, 370). While different species of clinical interest have been identified, including *S. globosa, S. brasiliensis, S. Mexicana*, and *S. luriei*, the most commonly reported species in human clinical isolates is *Sporothrix schenckii* (371, 372). Infection results in cutaneous or subcutaneous lesions usually with compromised adjacent lymphatic vessels. Rarely, disseminated disease can also ensue and several publications report infection of the lung, the CNS, bones, and other organs, mostly in immunocompromised individuals (372–375).

In a rat co-infection model of *Tenia taeniaeformis* and *S. schenckii*, a high expression of Dectin-1 only occurs in cutaneous lesions of co-infected rats, but is dispensable for the clearance of *S. schenckii* (376). However, a more recent study shows an increased Dectin-1 expression in peritoneal macrophages from *S. schenckii-*infected mice. Furthermore, the antibody-mediated blockade of Dectin-1 inhibits the cytokine production in response to different stimuli by peritoneal macrophages. A Dectin-1 blockade additionally results in a decreased phagocytic uptake of *S. schenckii* yeast cells (32). Martínez-Álvarez et al. proved that Dectin-1 is crucial for the secretion of cytokines by human PBMCs during *S. schenckii* infection, but dispensable for the recognition of *S. brasiliensis*. The authors also reported that while MR appears to have only a minor role in the recognition of *S. schenckii* yeast-like cells, it mediates the production of proinflammatory cytokines by human PBMCs in response to conidia from this fungus as well as yeasts from *S. brasiliensis* (47). An earlier study on the contribution of MR in the recognition of *S. schenckii* evidenced the presence of mannose residues in the cell wall of *S. schenckii* conidia and yeasts. Nevertheless, MR seemed to be involved only in the phagocytosis of opsonized conidia (377). Taken together, Dectin-1 seems to be important for the generation of cytokines while MR mainly plays a role in the phagocytosis of this fungus.

## Challenges and Future Directions

C-type lectin receptors recognize carbohydrate ligands in fungal cell walls and years of research have provided a huge body of evidence on their importance in modulating immune responses against fungal infections. However, there are still a number of gaps to be filled and areas that need attention from the scientific community. Almost all of the fungal infecitons are opportunistic, mainly concerning people with a compromised immune system, including HIV-infected individuals. Although they are a major high-risk group, many studies exclude HIV-infected patients and, therefore, this area of research seems to be largely unexplored. More studies focusing on HIV co-infection and the genetic make-up of HIV-infected individuals which affects their susceptibility toward developing specific fungal infections would be fundamental in developing tailored therapies for such cases. Fungi such as *Aspergillus* and *Candida* species interact with a number of CLRs upon infection, opening up opportunities to study the interactions between these receptors on a functional level [for example, the formation of heteromers as precdicted in case of Mincle and MCL (378)] as well as on a genetic level, including epistatic effects that the concerned genes have on each other. Another complex aspect that still needs to be studied in detail is how different cell types interact in an *in vivo* environment in order to control the infection. On the other hand, there are many fungal species such as *Paracoccidiodes, Fusarium*, etc., with few identified immune receptors, ligands, and/or virulence factors. Discovering these ligands and their corresponding receptors or new virulence factors will not only improve our understanding of fungal interactions with immune cells but also aid in developing vaccines and diagnostic or even therapeutic strategies against these fungi. Moreover, we now also know that CLRs not only recognize carbohydrate ligands but also lipids, proteins, and nucleic acids (3). We need to widen our views and explore this aspect more fully, since the fungal cell walls in addition to their manyfold carbohydrate structures also contain a number of lipids and proteins that may as well serve as potential ligands for CLRs (379). Moreover, some recent studies have demonstrated how differential immune responses are generated by different fungal isolates or strains depending on their individual pahogenic potential and virulence (380–383). Such studies suggest that a strain-specific comparison of immune response might be essential to fully understand the host–pathogen interactions. This knowledge would be helpful in generating data for individual case-specific therapies and, therefore, such studies need to be encouraged. Furthermore, it is often noted that many studies, such as *in vivo* mouse studies, provide contrasting results that lead to contradictions and questions. Therefore, the scientific studies need to be designed more carefully taking into account the strains of mice and the fungal species being studied, cell type or cell lines being used, and the type of *in vitro* or *in vivo* environment provided. Also, population structures and stratification in genetic studies should be addressed. The effects of each of these factors on the outcome of the results should be appropriately discussed in order to have a comprehensive view. In summary, there is a need to further enhance the understanding of how CLRs recognize pathogenic fungi in order to promote approaches to take advantage of this knowledge for future therapeutic interventions. The ability of these receptors to bind a wide variety of pathogens which share the same ligand as fungi, makes them important molecules in the innate immune response. While there are many functional aspects of CLRs yet to be discovered, there is an increasing amount of information published over the past decade that already allows us to benefit from our knowledge on regulatory functions and new translational opportunities.

## Conclusion

It is widely excepted that CLRs play a major role in modulating immune responses with respect to fungal infections, being able to recognize the carbohydrate moieties in the fungal cell walls. Fungal infections, though mainly opportunistic, can prove fatal in case of faulty diagnosis or treatment. The fungal recognition by CLRs mainly leads to proinflammatory responses and a subsequent activation of adaptive immunity *via* Th17 responses. However, negative or anti-inflammatory effects have also been noted and both types of responses are necessary to mount a specific immune response. A considerable body of work has been done with regards to frequent pathogens, such as *Candida, Aspergillus*, and *Cryptococcus*, etc., and genetic susceptibilities pertaining to fungal infections have been attributed to various mutations in CLRs. While some fungal infections are frequent, others are emerging into a major health problem with the continous increase in immuno-compromised patients. A thorough knowledge of the molecular mechanisms of fungal infections and the interaction of these fungi with their major receptors, the CLRs, can provide a basis to a better and more specific diagnosis and treatment regime. Also, knowledge about the host-related genetic factors, which can greatly affect the course and outcome of these infections, may advance a timely diagnosis and care for the patient.

## Author Contributions

HS conceived the review framework. SG and JC-B wrote the manuscript. EK and HS revised the manuscript. JC-B and SG created the figure. All authors have read and approved the final version of the manuscript.

## Conflict of Interest Statement

The authors declare that the research was conducted in the absence of any commercial or financial relationships that could be construed as a potential conflict of interest.

## References

[1] Plato A Hardison SE Brown GD. Pattern recognition receptors in antifungal immunity. Semin Immunopathol (2015) 37(2):97–106. 10.1007/s00281-014-0462-4 25420452 PMC4326652

[2] Geijtenbeek TBH Gringhuis SI. Signalling through C-type lectin receptors: shaping immune responses. Nat Rev Immunol (2009) 9(7):465–79. 10.1038/nri2569 19521399 PMC7097056

[3] Zelensky AN Gready JE. The C-type lectin-like domain superfamily. FEBS J (2005) 272(24):6179–217. 10.1111/j.1742-4658.2005.05031.x 16336259

[4] Drummond RA Gaffen SL Hise AG Brown GD Innate defense against fungal pathogens. Cold Spring Harb Perspect Med (2015) 5(6). 10.1101/cshperspect.a019620 PMC4426252 25384766

[5] Goyal S Klassert TE Slevogt H. C-type lectin receptors in tuberculosis: what we know. Med Microbiol Immunol (2016) 205(6):513–35. 10.1007/s00430-016-0470-1 27469378

[6] Drickamer K C-type lectin-like domains. Curr Opin Struct Biol (1999) 9(5):585–90. 10.1016/S0959-440X(99)00009-3 10508765

[7] Hoving JC Wilson GJ Brown GD. Signalling C-type lectin receptors, microbial recognition and immunity. Cell Microbiol (2014) 16(2):185–94. 10.1111/cmi.12249 24330199 PMC4016756

[8] Kerrigan AM Brown GD. Syk-coupled C-type lectin receptors that mediate cellular activation via single tyrosine based activation motifs. Immunol Rev (2010) 234:335–52. 10.1111/j.0105-2896.2009.00882.x 20193029

[9] Drummond RA Lionakis MS Mechanistic insights into the role of C-type lectin receptor/CARD9 signaling in human antifungal immunity. Front Cell Infect Microbiol (2016) 6:39 10.3389/fcimb.2016.00039 27092298 PMC4820464

[10] Yamasaki S Ishikawa E Sakuma M Hara H Ogata K Saito T. Mincle is an ITAM-coupled activating receptor that senses damaged cells. Nat Immunol (2008) 9(10):1179–88. 10.1038/ni.1651 18776906

[11] Sato K Yang XL Yudate T Chung JS Wu JM Luby-Phelps K Dectin-2 is a pattern recognition receptor for fungi that couples with the Fc receptor gamma chain to induce innate immune responses. J Biol Chem (2006) 281(50):38854–66. 10.1074/jbc.M606542200 17050534

[12] Heyl KA Klassert TE Heinrich A Müller MM Klaile E Dienemann H Dectin-1 is expressed in human lung and mediates the proinflammatory immune response to nontypeable *Haemophilus influenzae*. MBio (2014) 5:e1492–1414. 10.1128/mBio.01492-14 PMC4173778 25161190

[13] Isakov N ITIMs and ITAMs – the Yin and Yang of antigen and Fc receptor-linked signaling machinery. Immunol Res (1997) 16(1):85–100. 10.1007/BF02786325 9048210

[14] Richard M Thibault N Veilleux P Gareau-Page G Beaulieu AD. Granulocyte macrophage-colony stimulating factor reduces the affinity of SHP-2 for the ITIM of CLECSF6 in neutrophils: a new mechanism of action for SHP-2. Mol Immunol (2006) 43(10):1716–21. 10.1016/j.molimm.2005.10.006 16360206

[15] Khan NS Kasperkovitz PV Timmons AK Mansour MK Tam JM Seward MW Dectin-1 controls TLR9 trafficking to phagosomes containing β-1,3 glucan. J Immunol (2016) 196:2249–61. 10.4049/jimmunol.1401545 26829985 PMC4761466

[16] Sancho D Reis e Sousa C. Signaling by myeloid C-type lectin receptors in immunity and homeostasis. Annu Rev Immunol (2012) 30:491–529. 10.1146/annurev-immunol-031210-101352 22224766 PMC4480235

[17] Gantner BN Simmons RM Underhill DM. Dectin-1 mediates macrophage recognition of *Candida albicans* yeast but not filaments. EMBO J (2005) 24(6):1277–86. 10.1038/sj.emboj.7600594 15729357 PMC556398

[18] Giles SS Dagenais TRT Botts MR Keller NP Hull CM. Elucidating the pathogenesis of spores from the human fungal pathogen *Cryptococcus neoformans*. Infect Immun (2009) 77:3491–500. 10.1128/IAI.00334-09 19451235 PMC2715683

[19] Rapaka RR Goetzman ES Zheng M Vockley J McKinley L Kolls JK Enhanced defense against *Pneumocystis carinii* mediated by a novel Dectin-1 receptor Fc fusion protein. J Immunol (2007) 178:3702–12. 10.4049/jimmunol.178.6.3702 17339468

[20] Viriyakosol S Fierer J Brown GD Kirkland TN. Innate immunity to the pathogenic fungus *Coccidioides posadasii* is dependent on toll-like receptor 2 and Dectin-1. Infect Immun (2005) 73:1553–60. 10.1128/IAI.73.3.1553-1560.2005 15731053 PMC1064940

[21] Lin J-S Huang J-H Hung L-Y Wu S-Y Wu-Hsieh BA Distinct roles of complement receptor 3, Dectin-1, and sialic acids in murine macrophage interaction with *Histoplasma* yeast. J Leukoc Biol (2010) 88:95–106. 10.1189/jlb.1109717 20360401

[22] Bonfim CV Mamoni RL Lima Blotta MHS. TLR-2, TLR-4 and Dectin-1 expression in human monocytes and neutrophils stimulated by *Paracoccidioides brasiliensis*. Med Mycol (2009) 47:722–33. 10.3109/13693780802641425 19888805

[23] Nakamura K Miyazato A Koguchi Y Adachi Y Ohno N Saijo S Toll-like receptor 2 (TLR2) and Dectin-1 contribute to the production of IL-12p40 by bone marrow-derived dendritic cells infected with *Penicillium marneffei*. Microbes Infect (2008) 10:1223–7. 10.1016/j.micinf.2008.06.011 18652908

[24] Nakamura T Nishibu A Yoshida N Yasoshima M Anzawa K Watanabe Y Glycyrrhetinic acid inhibits contact hypersensitivity induced by trichophytin via Dectin-1. Exp Dermatol (2016) 25:299–304. 10.1111/exd.12931 26739065

[25] Kistowska M Fenini G Jankovic D Feldmeyer L Kerl K Bosshard P *Malassezia* yeasts activate the NLRP3 inflammasome in antigen-presenting cells via Syk-kinase signalling. Exp Dermatol (2014) 23:884–9. 10.1111/exd.12552 25267545

[26] Siqueira IM de Castro RJA Leonhardt LCDM Jerônimo MRS Soares AZC Raiol T Modulation of the immune response by *Fonsecaea pedrosoi* morphotypes in the course of experimental chromoblastomycosis and their role on inflammatory response chronicity. PLoS Negl Trop Dis (2017) 11:e0005461. 10.1371/journal.pntd.0005461 28355277 PMC5391973

[27] Yeh CC Horng HC Chou H Tai HY Shen HD Hsieh SL Dectin-1-mediated pathway contributes to *Fusarium proliferatum*-induced CXCL-8 release from human respiratory epithelial cells. Int J Mol Sci (2017) 18(3). 10.3390/ijms18030624 PMC5372638 28335387

[28] Higashino-Kameda M Yabe-Wada T Matsuba S Takeda K Anzawa K Mochizuki T A critical role of Dectin-1 in hypersensitivity pneumonitis. Inflamm Res (2016) 65:235–44. 10.1007/s00011-015-0910-1 26644324

[29] Graham LM Tsoni SV Willment JA Williams DL Taylor PR Gordon S Soluble Dectin-1 as a tool to detect β-glucans. J Immunol Methods (2006) 314:164–9. 10.1016/j.jim.2006.05.013 16844139

[30] Reedy JL Negoro PE Feliu M Lord AK Khan NS Lukason DP The carbohydrate lectin receptor Dectin-1 mediates the immune response to *Exserohilum rostratum*. Infect Immun (2017) 85:e903–16. 10.1128/IAI.00903-16 28031265 PMC5328484

[31] Mintz-Cole RA Brandt EB Bass SA Gibson AM Reponen T Hershey GKK. Surface availability of beta-glucans is critical determinant of host immune response to *Cladosporium cladosporioides*. J Allergy Clin Immunol (2013) 132(1):159–69. 10.1016/j.jaci.2013.01.003 23403046 PMC6145803

[32] Jellmayer JA Ferreira LS Manente FA Goncalves AC Polesi MC Batista-Duharte A Dectin-1 expression by macrophages and related antifungal mechanisms in a murine model of *Sporothrix schenckii* sensu stricto systemic infection. Microb Pathog (2017) 110:78–84. 10.1016/j.micpath.2017.06.025 28645771

[33] McGreal EP Rosas M Brown GD Zamze S Wong SYC Gordon S The carbohydrate-recognition domain of Dectin-2 is a C-type lectin with specificity for high mannose. Glycobiology (2006) 16:422–30. 10.1093/glycob/cwj077 16423983

[34] Saijo S Ikeda S Yamabe K Kakuta S Ishigame H Akitsu A Dectin-2 recognition of alpha-mannans and induction of Th17 cell differentiation is essential for host defense against *Candida albicans*. Immunity (2010) 32(5):681–91. 10.1016/j.immuni.2010.05.001 20493731

[35] Wang H LeBert V Hung CY Galles K Saijo S Lin X C-Type lectin receptors differentially induce Th17 cells and vaccine immunity to the endemic mycosis of North America. J Immunol (2014) 192:1107–19. 10.4049/jimmunol.1302314 24391211 PMC3910401

[36] Ishikawa T Itoh F Yoshida S Saijo S Matsuzawa T Gonoi T Identification of distinct ligands for the C-type lectin receptors mincle and Dectin-2 in the pathogenic fungus *Malassezia*. Cell Host Microbe (2013) 13:477–88. 10.1016/j.chom.2013.03.008 23601109

[37] Wüthrich M Wang H Li M Lerksuthirat T Hardison SE Brown GD *Fonsecaea pedrosoi*-induced Th17-cell differentiation in mice is fostered by Dectin-2 and suppressed by Mincle recognition. Eur J Immunol (2015) 45:2542–52. 10.1002/eji.201545591 26140582 PMC4562893

[38] Wang Q Zhao G Lin J Li C Jiang N Xu Q Role of the mannose receptor during *Aspergillus fumigatus* infection and interaction with Dectin-1 in corneal epithelial cells. Cornea (2016) 35:267–73. 10.1097/ICO.0000000000000710 26606296

[39] Cambi A Netea MG Mora-Montes HM Gow NA Hato SV Lowman DW Dendritic cell interaction with *Candida albicans* critically depends on N-linked mannan. J Biol Chem (2008) 283(29):20590–9. 10.1074/jbc.M709334200 18482990 PMC2459306

[40] Szolnoky G Bata-Csorgo Z Kenderessy AS Kiss M Pivarcsi A Novak Z A mannose-binding receptor is expressed on human keratinocytes and mediates killing of *Candida albicans*. J Invest Dermatol (2001) 117(2):205–13. 10.1046/j.1523-1747.2001.14071.x 11511295

[41] Neumann AK Jacobson K A novel pseudopodial component of the dendritic cell anti-fungal response: the fungipod. PLoS Pathog (2010) 6(2):e1000760 10.1371/journal.ppat.1000760 20169183 PMC2820528

[42] Mansour MK Schlesinger LS Levitz SM. Optimal T cell responses to *Cryptococcus neoformans* mannoprotein are dependent on recognition of conjugated carbohydrates by mannose receptors. J Immunol (2002) 168:2872–9. 10.4049/jimmunol.168.6.2872 11884457

[43] Zhang J Zhu J Imrich A Cushion M Kinane TB Koziel H. *Pneumocystis* activates human alveolar macrophage NF-kappaB signaling through mannose receptors. Infect Immun (2004) 72:3147–60. 10.1128/IAI.72.6.3147-3160.2004 15155616 PMC415687

[44] Nakaira-Takahagi E Golim MA Bannwart CF Puccia R Peraçoli MTS. Interactions between TLR2, TLR4, and mannose receptors with gp43 from *Paracoccidioides brasiliensis* induce cytokine production by human monocytes. Med Mycol (2011) 49:1–10. 10.3109/13693786.2011.565485 21417682

[45] Koguchi Y Kawakami K Kon S Segawa T Maeda M Uede T *Penicillium marneffei* causes osteopontin-mediated production of interleukin-12 by peripheral blood mononuclear cells. Infect Immun (2002) 70(3):1042–8. 10.1128/IAI.70.3.1042-1048.2002 11854181 PMC127744

[46] Giaimis J Lombard Y Fonteneau P Muller CD Levy R Makaya-Kumba M Both mannose and beta-glucan receptors are involved in phagocytosis of unopsonized, heat-killed *Saccharomyces cerevisiae* by murine macrophages. J Leukoc Biol (1993) 54:564–71. 10.1002/jlb.54.6.564 8245708

[47] Martinez-Alvarez JA Perez-Garcia LA Mellado-Mojica E Lopez MG Martinez-Duncker I Lopes-Bezerra LM *Sporothrix schenckii* sensu stricto and *Sporothrix brasiliensis* are differentially recognized by human peripheral blood mononuclear cells. Front Microbiol (2017) 8:843. 10.3389/fmicb.2017.00843 28539922 PMC5423980

[48] Allen MJ Voelker DR Mason RJ. Interactions of surfactant proteins A and D with *Saccharomyces cerevisiae* and *Aspergillus fumigatus*. Infect Immun (2001) 69:2037–44. 10.1128/IAI.69.4.2037-2044.2001 11254556 PMC98128

[49] Schelenz S Malhotra R Sim RB Holmskov U Bancroft GJ. Binding of host collectins to the pathogenic yeast *Cryptococcus neoformans*: human surfactant protein D acts as an agglutinin for acapsular yeast cells. Infect Immun (1995) 63:3360–6. 7642263 10.1128/iai.63.9.3360-3366.1995 PMC173462

[50] van de Wetering JK Coenjaerts FEJ Vaandrager AB van Golde LMG Batenburg JJ. Aggregation of *Cryptococcus neoformans* by surfactant protein D is inhibited by its capsular component glucuronoxylomannan. Infect Immun (2004) 72:145–53. 10.1128/IAI.72.1.145-153.2004 14688091 PMC343972

[51] Vuk-Pavlovic Z Standing JE Crouch EC Limper AH. Carbohydrate recognition domain of surfactant protein D mediates interactions with *Pneumocystis carinii* glycoprotein A. Am J Respir Cell Mol Biol (2001) 24:475–84. 10.1165/ajrcmb.24.4.3504 11306442

[52] McCormack FX Festa AL Andrews RP Linke M Walzer PD The carbohydrate recognition domain of surfactant protein A mediates binding to the major surface glycoprotein of *Pneumocystis carinii*. Biochemistry (1997) 36:8092–9. 10.1021/bi970313f 9201957

[53] Awasthi S Magee DM Coalson JJ. *Coccidioides posadasii* infection alters the expression of pulmonary surfactant proteins (SP)-A and SP-D. Respir Res (2004) 5:28. 10.1186/1465-9921-5-28 15588319 PMC543449

[54] McCormack FX Gibbons R Ward SR Kuzmenko A Wu H Deepe GS. Macrophage-independent fungicidal action of the pulmonary collectins. J Biol Chem (2003) 278:36250–6. 10.1074/jbc.M303086200 12857753

[55] Neth O Jack DL Dodds AW Holzel H Klein NJ Turner MW. Mannose-binding lectin binds to a range of clinically relevant microorganisms and promotes complement deposition. Infect Immun (2000) 68:688–93. 10.1128/IAI.68.2.688-693.2000 10639434 PMC97193

[56] Lillegard JB Sim RB Thorkildson P Gates MA Kozel TR. Recognition of *Candida albicans* by mannan-binding lectin in vitro and in vivo. J Infect Dis (2006) 193(11):1589–97. 10.1086/503804 16652289

[57] Mershon-Shier KL Vasuthasawat A Takahashi K Morrison SL Beenhouwer DO. In vitro C3 deposition on cryptococcus capsule occurs via multiple complement activation pathways. Mol Immunol (2011) 48:2009–18. 10.1016/j.molimm.2011.06.215 21723612 PMC3163710

[58] Laursen AL Obel N Holmskov U Jensenius JC Aliouat EM Andersen PL. Activation of the respiratory burst by *Pneumocystis carinii*. Efficiency of different antibody isotypes, complement, lung surfactant protein D, and mannan-binding lectin. APMIS (2003) 111:405–15. 10.1034/j.1600-0463.2003.t01-1-1110205.x 12752220

[59] Serrano-Gómez D Leal JA Corbí AL DC-SIGN mediates the binding of *Aspergillus fumigatus* and keratinophylic fungi by human dendritic cells. Immunobiology (2005) 210:175–83. 10.1016/j.imbio.2005.05.011 16164024

[60] Serrano-Gómez D Domínguez-Soto A Ancochea J Jimenez-Heffernan JA Leal JA Corbí AL. Dendritic cell-specific intercellular adhesion molecule 3-grabbing nonintegrin mediates binding and internalization of *Aspergillus fumigatus* conidia by dendritic cells and macrophages. J Immunol (2004) 173:5635–43. 10.4049/jimmunol.173.9.5635 15494514

[61] Cambi A Gijzen K de Vries IJM Torensma R Joosten B Adema GJ The C-type lectin DC-SIGN (CD209) is an antigen-uptake receptor for *Candida albicans* on dendritic cells. Eur J Immunol (2003) 33:532–8. 10.1002/immu.200310029 12645952

[62] Mansour MK Latz E Levitz SM *Cryptococcus neoformans* glycoantigens are captured by multiple lectin receptors and presented by dendritic cells. J Immunol (2006) 176:3053–61. 10.4049/jimmunol.176.5.3053 16493064

[63] Ngaosuwankul P Pongtanalert P Engering A Chaiyaroj SC. Differential gene expression profiles of human monocyte-derived antigen presenting cells in response to *Penicillium marneffei*: roles of DC-SIGN (CD209) in fungal cell uptake. Asian Pac J Allergy Immunol (2008) 26(2–3):151–63. 19054934

[64] Takahara K Arita T Tokieda S Shibata N Okawa Y Tateno H Difference in fine specificity to polysaccharides of *Candida albicans* mannoprotein between mouse SIGNR1 and human DC-SIGN. Infect Immun (2012) 80:1699–706. 10.1128/IAI.06308-11 22331432 PMC3347427

[65] Zhao G Xu Q Lin J Chen W Cui T Hu L The role of Mincle in innate immune to fungal keratitis. J Infect Dev Ctries (2017) 11:89–97. 10.3855/jidc.7570 28141595

[66] Wells CA Salvage-Jones JA Li X Hitchens K Butcher S Murray RZ The macrophage-inducible C-type lectin, Mincle, is an essential component of the innate immune response to *Candida albicans*. J Immunol (2008) 180(11):7404–13. 10.4049/jimmunol.180.11.7404 18490740

[67] Kottom TJ Hebrink DM Jenson PE Nandakumar V Wüthrich M Wang H The interaction of *Pneumocystis* with the C-type lectin receptor mincle exerts a significant role in host defense against infection. J Immunol (2017) 198:3515–25. 10.4049/jimmunol.1600744 28298521 PMC5423441

[68] da Glória Sousa M Reid DM Schweighoffer E Tybulewicz V Ruland JR Langhorne J Restoration of pattern recognition receptor costimulation to treat chromoblastomycosis, a chronic fungal infection of the skin. Cell Host Microbe (2011) 9:436–43. 10.1016/j.chom.2011.04.005 21575914 PMC3098964

[69] Kerscher B Willment JA Brown GD. The Dectin-2 family of C-type lectin-like receptors: an update. Int Immunol (2013) 25(5):271–7. 10.1093/intimm/dxt006 23606632 PMC3631001

[70] Zhu LL Zhao XQ Jiang C You Y Chen XP Jiang YY C-type lectin receptors Dectin-3 and Dectin-2 form a heterodimeric pattern-recognition receptor for host defense against fungal infection. Immunity (2013) 39:324–34. 10.1016/j.immuni.2013.05.017 23911656

[71] Hole CR Leopold Wager CM Mendiola AS Wozniak KL Campuzano A Lin X Antifungal activity of plasmacytoid dendritic cells against *Cryptococcus neoformans* in vitro requires expression of Dectin-3 (CLEC4D) and reactive oxygen species. Infect Immun (2016) 84:2493–504. 10.1128/IAI.00103-16 27324480 PMC4995896

[72] Gresnigt MS Becker KL Smeekens SP Jacobs CWM Joosten LAB van der Meer JWM *Aspergillus fumigatus*-induced IL-22 is not restricted to a specific Th cell subset and is dependent on complement receptor 3. J Immunol (2013) 190:5629–39. 10.4049/jimmunol.1202601 23645883

[73] Forsyth CB Plow EF Zhang L. Interaction of the fungal pathogen *Candida albicans* with integrin CD11b/CD18: recognition by the I domain is modulated by the lectin-like domain and the CD18 subunit. J Immunol (1998)161(11):6198–205. 9834106

[74] Soloviev DA Fonzi WA Sentandreu R Pluskota E Forsyth CB Yadav S Identification of pH-regulated antigen 1 released from *Candida albicans* as the major ligand for leukocyte integrin alphaMbeta2. J Immunol (2007) 178(4):2038–46. 10.4049/jimmunol.178.4.2038 17277107

[75] Huang J-H Lin C-Y Wu S-Y Chen W-Y Chu C-L Brown GD CR3 and Dectin-1 collaborate in macrophage cytokine response through association on lipid rafts and activation of Syk-JNK-AP-1 pathway. PLoS Pathog (2015) 11:e1004985. 10.1371/journal.ppat.1004985 26132276 PMC4488469

[76] Jiménez Mdel P Restrepo A Radzioch D Cano LE García LF. Importance of complement 3 and mannose receptors in phagocytosis of *Paracoccidioides brasiliensis* conidia by Nramp1 congenic macrophages lines. FEMS Immunol Med Microbiol (2006) 47:56–66. 10.1111/j.1574-695X.2006.00059.x 16706788

[77] Suzuki T Ohno N Ohshima Y Yadomae T Soluble mannan and Î^2^-glucan inhibit the uptake of *Malassezia furfur* by human monocytic cell line, THP-1. FEMS Immunol Med Microbiol (1998) 21:223–30. 10.1111/j.1574-695X.1998.tb01169.x 9718212

[78] van Bruggen R Drewniak A Jansen M van Houdt M Roos D Chapel H Complement receptor 3, not Dectin-1, is the major receptor on human neutrophils for β-glucan-bearing particles. Mol Immunol (2009) 47:575–81. 10.1016/j.molimm.2009.09.018 19811837

[79] Gao X Zhao G Li C Lin J Jiang N Wang Q LOX-1 and TLR4 affect each other and regulate the generation of ROS in *A. fumigatus* keratitis. Int Immunopharmacol (2016) 40:392–9. 10.1016/j.intimp.2016.09.027 27694040

[80] Li C Zhao G Che C Lin J Li N Hu L The role of LOX-1 in innate immunity to *Aspergillus fumigatus* in corneal epithelial cells. Invest Opthalmol Vis Sci (2015) 56:3593. 10.1167/iovs.14-15989 26047046

[81] de Jong MA Vriend LE Theelen B Taylor ME Fluitsma D Boekhout T C-type lectin Langerin is a beta-glucan receptor on human Langerhans cells that recognizes opportunistic and pathogenic fungi. Mol Immunol (2010) 47:1216–25. 10.1016/j.molimm.2009.12.016 20097424 PMC2837148

[82] Stappers MHT Clark AE Aimanianda V Bidula S Reid DM Asamaphan P Recognition of DHN-melanin by a C-type lectin receptor is required for immunity to *Aspergillus*. Nature (2018) 555(7696):382–6. 10.1038/nature25974 29489751 PMC5857201

[83] Havlickova B Czaika VA Friedrich M. Epidemiological trends in skin mycoses worldwide. Mycoses (2008) 51(Suppl 4):2–15. 10.1111/j.1439-0507.2008.01606.x 18783559

[84] Brown GD Denning DW Gow NA Levitz SM Netea MG White TC Hidden killers: human fungal infections. Sci Transl Med (2012) 4(165):165rv13 10.1126/scitranslmed.3004404 23253612

[85] Kim JY Human fungal pathogens: Why should we learn? J Microbiol (2016) 54:145–8. 10.1007/s12275-016-0647-8 26920875

[86] Gow NAR Latge JP Munro CA. The fungal cell wall: structure, biosynthesis, and function. Microbiol Spectr (2017) 5(3). 10.1128/microbiolspec.FUNK-0035-2016 28513415 PMC11687499

[87] Warris A The biology of pulmonary *Aspergillus* infections. J Infect (2014) 69:S36–41. 10.1016/j.jinf.2014.07.011 25135079

[88] Loures FV Röhm M Lee CK Santos E Wang JP Specht CA Recognition of *Aspergillus fumigatus* hyphae by human plasmacytoid dendritic cells is mediated by Dectin-2 and results in formation of extracellular traps. PLoS Pathog (2015) 11:e1004643. 10.1371/journal.ppat.1004643 25659141 PMC4450068

[89] Gersuk GM Underhill DM Zhu L Marr KA. Dectin-1 and TLRs permit macrophages to distinguish between different *Aspergillus fumigatus* cellular states. J Immunol (2006) 176:3717–24. 10.4049/jimmunol.176.6.3717 16517740

[90] Sun H Xu X-Y Shao H-T Su X Wu X-D Wang Q Dectin-2 is predominately macrophage restricted and exhibits conspicuous expression during *Aspergillus fumigatus* invasion in human lung. Cell Immunol (2013) 284:60–7. 10.1016/j.cellimm.2013.06.013 23928558

[91] Madan T Eggleton P Kishore U Strong P Aggrawal SS Sarma PU Binding of pulmonary surfactant proteins A and D to *Aspergillus fumigatus* conidia enhances phagocytosis and killing by human neutrophils and alveolar macrophages. Infect Immun (1997) 65:3171–9. 9234771 10.1128/iai.65.8.3171-3179.1997 PMC175448

[92] Steele C Rapaka RR Metz A Pop SM Williams DL Gordon S The beta-glucan receptor Dectin-1 recognizes specific morphologies of *Aspergillus fumigatus*. PLoS Pathog (2005) 1:e42. 10.1371/journal.ppat.0010042 16344862 PMC1311140

[93] Hohl TM Van Epps HL Rivera A Morgan LA Chen PL Feldmesser M *Aspergillus fumigatus* triggers inflammatory responses by stage-specific β-glucan display. PLoS Pathog (2005) 1:e30 10.1371/journal.ppat.0010030 16304610 PMC1287910

[94] Lamoth F Rubino I Bochud P-Y. Immunogenetics of invasive aspergillosis. Med Mycol (2011) 49:S125–36. 10.3109/13693786.2010.516408 20840014

[95] Gravelat FN Beauvais A Liu H Lee MJ Snarr BD Chen D *Aspergillus* galactosaminogalactan mediates adherence to host constituents and conceals hyphal β-glucan from the immune system. PLoS Pathog (2013) 9:e1003575 10.1371/journal.ppat.1003575 23990787 PMC3749958

[96] Savers A Rasid O Parlato M Brock M Jouvion G Ryffel B Infection-mediated priming of phagocytes protects against lethal secondary *Aspergillus fumigatus* challenge. PLoS One (2016) 11:e0153829. 10.1371/journal.pone.0153829 27078879 PMC4831689

[97] Yang J-X Liu W Lu Q-Y Wan Z Wang X-H Li R-Y. Different expression of Dectin-1 and toll-like receptor 2 in the lungs of different immune status mice infected with *Aspergillus fumigatus*. Chin Med J(Engl) (2009) 122:2017–21. 19781388

[98] Werner JL Metz AE Horn D Schoeb TR Hewitt MM Schwiebert LM Requisite role for the Dectin-1-glucan receptor in pulmonary defense against *Aspergillus fumigatus*. J Immunol (2009) 182:4938–46. 10.4049/jimmunol.0804250 19342673 PMC3434356

[99] Mezger M Kneitz S Wozniok I Kurzai O Einsele H Loeffler J. Proinflammatory response of immature human dendritic cells is mediated by Dectin-1 after exposure to *Aspergillus fumigatus* germ tubes. J Infect Dis (2008) 197:924–31. 10.1086/528694 18279049

[100] Wang M Liu Z Liu C Wu T Cai F Wang Q PU.1 is involved in the immune response to *Aspergillus fumigatus* through upregulating Dectin-1 expression. BMC Infect Dis (2016) 16:297. 10.1186/s12879-016-1632-x 27306059 PMC4910222

[101] Serezani CH Kane S Collins L Morato-Marques M Osterholzer JJ Peters-Golden M. Macrophage Dectin-1 expression is controlled by leukotriene B4 via a GM-CSF/PU.1 axis. J Immunol (2012) 189:906–15. 10.4049/jimmunol.1200257 22696442 PMC3392366

[102] Toyotome T Adachi Y Watanabe A Ochiai E Ohno N Kamei K Activator protein 1 is triggered by *Aspergillus fumigatus* β-glucans surface-exposed during specific growth stages. Microb Pathog (2008) 44:141–50. 10.1016/j.micpath.2007.08.015 17928189

[103] Luther K Torosantucci A Brakhage AA Heesemann J Ebel F. Phagocytosis of *Aspergillus fumigatus* conidia by murine macrophages involves recognition by the Dectin-1 beta-glucan receptor and toll-like receptor 2. Cell Microbiol (2007) 9:368–81. 10.1111/j.1462-5822.2006.00796.x 16953804

[104] Camargo JF Bhimji A Kumar D Kaul R Pavan R Schuh A Impaired T cell responsiveness to interleukin-6 in hematological patients with invasive aspergillosis. PLoS One (2015) 10:e0123171. 10.1371/journal.pone.0123171 25835547 PMC4383538

[105] Xia D Sun W-K Tan M-M Ding Y Liu Z-C Li P An adenoviral vector encoding full-length Dectin-1 promotes *Aspergillus*-induced innate immune response in macrophages. Lung (2015) 193:549–57. 10.1007/s00408-015-9740-8 25944256

[106] Sun W-K Lu X Li X Sun Q-Y Su X Song Y Dectin-1 is inducible and plays a crucial role in *Aspergillus*-induced innate immune responses in human bronchial epithelial cells. Eur J Clin Microbiol Infect Dis (2012) 31:2755–64. 10.1007/s10096-012-1624-8 22562430

[107] Rivera A Hohl TM Collins N Leiner I Gallegos A Saijo S Dectin-1 diversifies *Aspergillus fumigatus*-specific T cell responses by inhibiting T helper type 1 CD4 T cell differentiation. J Exp Med (2011) 208:369–81. 10.1084/jem.20100906 21242294 PMC3039849

[108] Zhong J Huang W Deng Q Wu M Jiang H Lin X Inhibition of TREM-1 and Dectin-1 alleviates the severity of fungal keratitis by modulating innate immune responses. PLoS One (2016) 11:e0150114. 10.1371/journal.pone.0150114 26963514 PMC4786258

[109] Xu Q Zhao G Lin J Wang Q Hu L Jiang Z. Role of Dectin-1 in the innate immune response of rat corneal epithelial cells to *Aspergillus fumigatus*. BMC Ophthalmol (2015) 15:126. 10.1186/s12886-015-0112-1 26427623 PMC4591637

[110] Leal SM Cowden S Hsia Y-C Ghannoum MA Momany M Pearlman E. Distinct roles for Dectin-1 and TLR4 in the pathogenesis of *Aspergillus fumigatus* keratitis. PLoS Pathog (2010) 6:e1000976. 10.1371/journal.ppat.1000976 20617171 PMC2895653

[111] Sun H Xu X-Y Tian X-L Shao H-T Wu X-D Wang Q Activation of NF-κB and respiratory burst following *Aspergillus fumigatus* stimulation of macrophages. Immunobiology (2014) 219:25–36. 10.1016/j.imbio.2013.06.013 23886693

[112] Allen MJ Harbeck R Smith B Voelker DR Mason RJ. Binding of rat and human surfactant proteins A and D to *Aspergillus fumigatus* conidia. Infect Immun (1999) 67:4563–9. 10456901 10.1128/iai.67.9.4563-4569.1999 PMC96779

[113] Kaur S Gupta VK Thiel S Sarma PU Madan T. Protective role of mannan-binding lectin in a murine model of invasive pulmonary aspergillosis. Clin Exp Immunol (2007) 148:382–9. 10.1111/j.1365-2249.2007.03351.x 17335555 PMC1868875

[114] Geunes-Boyer S Heitman J Wright JR Steinbach WJ Surfactant protein D binding to *Aspergillus fumigatus* hyphae is calcineurin-sensitive. Med Mycol (2010) 48:580–8. 10.3109/13693780903401682 20141481 PMC3676939

[115] Singh M Madan T Waters P Sonar S Singh SK Kamran MF Therapeutic effects of recombinant forms of full-length and truncated human surfactant protein D in a murine model of invasive pulmonary aspergillosis. Mol Immunol (2009) 46:2363–9. 10.1016/j.molimm.2009.03.019 19403176

[116] Che C-Y Jia W-Y Xu Q Li N Hu L-T Jiang N The roles of surfactant protein D during *Aspergillus fumigatus* infection in human corneal epithelial cells. Int J Ophthalmol (2012) 5:13–7. 10.3980/j.issn.2222-3959.2012.01.03 22553747 PMC3340838

[117] Wu X Zhao G Lin J Jiang N Li C Hu L The production mechanism and immunosuppression effect of pulmonary surfactant protein D via toll like receptor 4 signaling pathway in human corneal epithelial cells during *Aspergillus fumigatus* infection. Int Immunopharmacol (2015) 29:433–9. 10.1016/j.intimp.2015.10.018 26507163

[118] Sainz J Lupiáñez CB Segura-Catena J Vazquez L Ríos R Oyonarte S Dectin-1 and DC-SIGN polymorphisms associated with invasive pulmonary aspergillosis infection. PLoS One (2012) 7:e32273. 10.1371/journal.pone.0032273 22384201 PMC3288082

[119] Cunha C Di Ianni M Bozza S Giovannini G Zagarella S Zelante T Dectin-1 Y238X polymorphism associates with susceptibility to invasive aspergillosis in hematopoietic transplantation through impairment of both recipient- and donor-dependent mechanisms of antifungal immunity. Blood (2010) 116:5394–402. 10.1182/blood-2010-04-279307 20807886

[120] Chai LYA de Boer MGJ van der Velden WJFM Plantinga TS van Spriel AB Jacobs C The Y238X stop codon polymorphism in the human β-glucan receptor Dectin-1 and susceptibility to invasive aspergillosis. J Infect Dis (2011) 203:736–43. 10.1093/infdis/jiq102 21242599 PMC3072717

[121] Qu X Che C Gao A Lin J Wang N Du X Association of Dectin-1 and DC-SIGN gene single nucleotide polymorphisms with fungal keratitis in the Northern Han Chinese population. Mol Vis (2015) 21:391–402. 25883525 PMC4392830

[122] Sainz J Segura-Catena J Jurado M. [Association between genetic polymorphism in the promotor region of CD209 and propensity to develop invasive pulmonary aspergillosis]. Methods Find Exp Clin Pharmacol (2010) 32(Suppl A):9–13. 21381282

[123] Madan T Potential of lung surfactant proteins, SP-A and SP-D, and mannan binding lectin for therapy and genetic predisposition to allergic and invasive aspergillosis. Recent Pat Inflamm Allergy Drug Discov (2007) 1:183–7. 10.2174/187221307782418874 19075981

[124] Madan T Kaur S Saxena S Singh M Kishore U Thiel S Role of collectins in innate immunity against aspergillosis. Med Mycol (2005) 43:155–63. 10.1080/13693780500088408 16114131

[125] Kaur S Gupta VK Shah A Thiel S Sarma PU Madan T Elevated levels of mannan-binding leptin (MBL) and eosinophilia in patients of bronchial asthma with allergic rhinitis and allergic bronchopulmonary aspergillosis associate with a novel intronic polymorphism in MBL. Clin Exp Immunol (2006) 143:414–9. 10.1111/j.1365-2249.2006.03007.x 16487239 PMC1809600

[126] Saxena S Madan T Shah A Muralidhar K Sarma PU. Association of polymorphisms in the collagen region of SP-A2 with increased levels of total IgE antibodies and eosinophilia in patients with allergic bronchopulmonary aspergillosis. J Allergy Clin Immunol (2003) 111:1001–7. 10.1067/mai.2003.1395 12743564

[127] Vaid M Kaur S Sambatakou H Madan T Denning DW Sarma PU. Distinct alleles of mannose-binding lectin (MBL) and surfactant proteins A (SP-A) in patients with chronic cavitary pulmonary aspergillosis and allergic bronchopulmonary aspergillosis. Clin Chem Lab Med (2007) 45:183–6. 10.1515/CCLM.2007.033 17311505

[128] Crosdale DJ Poulton KV Ollier WE Thomson W Denning DW. Mannose-binding lectin gene polymorphisms as a susceptibility factor for chronic necrotizing pulmonary aspergillosis. J Infect Dis (2001) 184(5):653–6. 10.1086/322791 11474427

[129] Gow NA van de Veerdonk FL Brown AJ Netea MG. *Candida albicans* morphogenesis and host defence: discriminating invasion from colonization. Nat Rev Microbiol (2011) 10(2):112–22. 10.1038/nrmicro2711 22158429 PMC3624162

[130] Calderone RA Fonzi WA Virulence factors of *Candida albicans*. Trends Microbiol (2001) 9(7):327–35. 10.1016/S0966-842X(01)02094-7 11435107

[131] Hani U Shivakumar HG Vaghela R Osmani RA Shrivastava A Candidiasis: a fungal infection – current challenges and progress in prevention and treatment. Infect Disord Drug Targets (2015) 15(1):42–52. 10.2174/1871526515666150320162036 25809621

[132] Brown GD Herre J Williams DL Willment JA Marshall AS Gordon S. Dectin-1 mediates the biological effects of beta-glucans. J Exp Med (2003) 197(9):1119–24. 10.1084/jem.20021890 12719478 PMC2193964

[133] Adachi Y Ishii T Ikeda Y Hoshino A Tamura H Aketagawa J Characterization of beta-glucan recognition site on C-type lectin, Dectin 1. Infect Immun (2004) 72(7):4159–71. 10.1128/IAI.72.7.4159-4171.2004 15213161 PMC427417

[134] Kashem SW Igyarto BZ Gerami-Nejad M Kumamoto Y Mohammed JA Jarrett E *Candida albicans* morphology and dendritic cell subsets determine T helper cell differentiation. Immunity (2015) 42(2):356–66. 10.1016/j.immuni.2015.01.008 25680275 PMC4343045

[135] Bain JM Louw J Lewis LE Okai B Walls CA Ballou ER *Candida albicans* hypha formation and mannan masking of beta-glucan inhibit macrophage phagosome maturation. MBio (2014) 5(6):e01874 10.1128/mBio.01874-14 25467440 PMC4324242

[136] Cheng SC van de Veerdonk FL Lenardon M Stoffels M Plantinga T Smeekens S The Dectin-1/inflammasome pathway is responsible for the induction of protective T-helper 17 responses that discriminate between yeasts and hyphae of *Candida albicans*. J Leukoc Biol (2011) 90(2):357–66. 10.1189/jlb.1210702 21531876 PMC3513931

[137] Gantner BN Simmons RM Canavera SJ Akira S Underhill DM. Collaborative induction of inflammatory responses by Dectin-1 and toll-like receptor 2. J Exp Med (2003) 197(9):1107–17. 10.1084/jem.20021787 12719479 PMC2193968

[138] Rogers NC Slack EC Edwards AD Nolte MA Schulz O Schweighoffer E Syk-dependent cytokine induction by Dectin-1 reveals a novel pattern recognition pathway for C type lectins. Immunity (2005) 22(4):507–17. 10.1016/j.immuni.2005.06.005 15845454

[139] Underhill DM Rossnagle E Lowell CA Simmons RM. Dectin-1 activates Syk tyrosine kinase in a dynamic subset of macrophages for reactive oxygen production. Blood (2005) 106(7):2543–50. 10.1182/blood-2005-03-1239 15956283 PMC1895265

[140] Gross O Gewies A Finger K Schafer M Sparwasser T Peschel C Card9 controls a non-TLR signalling pathway for innate anti-fungal immunity. Nature (2006) 442(7103):651–6. 10.1038/nature04926 16862125

[141] Cohen-Kedar S Baram L Elad H Brazowski E Guzner-Gur H Dotan I Human intestinal epithelial cells respond to beta-glucans via Dectin-1 and Syk. Eur J Immunol (2014) 44(12):3729–40. 10.1002/eji.201444876 25251945

[142] Gow NA Netea MG Munro CA Ferwerda G Bates S Mora-Montes HM Immune recognition of *Candida albicans* beta-glucan by Dectin-1. J Infect Dis (2007) 196(10):1565–71. 10.1086/523110 18008237 PMC2655640

[143] Smeekens SP Gresnigt MS Becker KL Cheng SC Netea SA Jacobs L An anti-inflammatory property of *Candida albicans* beta-glucan: induction of high levels of interleukin-1 receptor antagonist via a Dectin-1/CR3 independent mechanism. Cytokine (2015) 71(2):215–22. 10.1016/j.cyto.2014.10.013 25461401 PMC4437193

[144] Da Silva CA Chalouni C Williams A Hartl D Lee CG Elias JA. Chitin is a size-dependent regulator of macrophage TNF and IL-10 production. J Immunol (2009) 182(6):3573–82. 10.4049/jimmunol.0802113 19265136

[145] Singh A Lelis F Braig S Schafer I Hartl D Rieber N. Differential regulation of myeloid-derived suppressor cells by *Candida* species. Front Microbiol (2016) 7:1624. 10.3389/fmicb.2016.01624 27790210 PMC5061774

[146] de Turris V Teloni R Chiani P Bromuro C Mariotti S Pardini M *Candida albicans* targets a lipid raft/Dectin-1 platform to enter human monocytes and induce antigen specific T cell responses. PLoS One (2015) 10:e0142531. 10.1371/journal.pone.0142531 26562838 PMC4643028

[147] Smith IM Baker A Christensen JE Boekhout T Frokiaer H Arneborg N *Kluyveromyces marxianus* and *Saccharomyces boulardii* induce distinct levels of dendritic cell cytokine secretion and significantly different T cell responses in vitro. PLoS One (2016) 11(11):e0167410. 10.1371/journal.pone.0167410 27898740 PMC5127564

[148] Daley D Mani VR Mohan N Akkad N Ochi A Heindel DW Dectin 1 activation on macrophages by galectin 9 promotes pancreatic carcinoma and peritumoral immune tolerance. Nat Med (2017) 23(5):556–67. 10.1038/nm.4314 28394331 PMC5419876

[149] Strasser D Neumann K Bergmann H Marakalala MJ Guler R Rojowska A Syk kinase-coupled C-type lectin receptors engage protein kinase C-sigma to elicit Card9 adaptor-mediated innate immunity. Immunity (2012) 36(1):32–42. 10.1016/j.immuni.2011.11.015 22265677 PMC3477316

[150] Nieto-Patlan A Campillo-Navarro M Rodriguez-Cortes O Munoz-Cruz S Wong-Baeza I Estrada-Parra S Recognition of *Candida albicans* by Dectin-1 induces mast cell activation. Immunobiology (2015) 220(9):1093–100. 10.1016/j.imbio.2015.05.005 26001731

[151] Pinke KH Lima HG Cunha FQ Lara VS. Mast cells phagocyte *Candida albicans* and produce nitric oxide by mechanisms involving TLR2 and Dectin-1. Immunobiology (2016) 221(2):220–7. 10.1016/j.imbio.2015.09.004 26421959

[152] Netea MG Gow NA Munro CA Bates S Collins C Ferwerda G Immune sensing of *Candida albicans* requires cooperative recognition of mannans and glucans by lectin and toll-like receptors. J Clin Invest (2006) 116(6):1642–50. 10.1172/JCI27114 16710478 PMC1462942

[153] Ferwerda G Meyer-Wentrup F Kullberg BJ Netea MG Adema GJ. Dectin-1 synergizes with TLR2 and TLR4 for cytokine production in human primary monocytes and macrophages. Cell Microbiol (2008) 10(10):2058–66. 10.1111/j.1462-5822.2008.01188.x 18549457

[154] del Fresno C Soulat D Roth S Blazek K Udalova I Sancho D Interferon-beta production via Dectin-1-Syk-IRF5 signaling in dendritic cells is crucial for immunity to *C. albicans*. Immunity (2013) 38(6):1176–86. 10.1016/j.immuni.2013.05.010 23770228

[155] Jia XM Tang B Zhu LL Liu YH Zhao XQ Gorjestani S CARD9 mediates Dectin-1-induced ERK activation by linking Ras-GRF1 to H-Ras for antifungal immunity. J Exp Med (2014) 211(11):2307–21. 10.1084/jem.20132349 25267792 PMC4203953

[156] Kennedy AD Willment JA Dorward DW Williams DL Brown GD DeLeo FR. Dectin-1 promotes fungicidal activity of human neutrophils. Eur J Immunol (2007) 37(2):467–78. 10.1002/eji.200636653 17230442

[157] Maneu V Yanez A Murciano C Molina A Gil ML Gozalbo D. Dectin-1 mediates in vitro phagocytosis of *Candida albicans* yeast cells by retinal microglia. FEMS Immunol Med Microbiol (2011) 63(1):148–50. 10.1111/j.1574-695X.2011.00829.x 21668824

[158] Mansour MK Tam JM Khan NS Seward M Davids PJ Puranam S Dectin-1 activation controls maturation of beta-1,3-glucan-containing phagosomes. J Biol Chem (2013) 288(22):16043–54. 10.1074/jbc.M113.473223 23609446 PMC3668760

[159] Goodridge HS Simmons RM Underhill DM. Dectin-1 stimulation by *Candida albicans* yeast or zymosan triggers NFAT activation in macrophages and dendritic cells. J Immunol (2007) 178(5):3107–15. 10.4049/jimmunol.178.5.3107 17312158

[160] Gringhuis SI den Dunnen J Litjens M van der Vlist M Wevers B Bruijns SC Dectin-1 directs T helper cell differentiation by controlling noncanonical NF-kappaB activation through Raf-1 and Syk. Nat Immunol (2009) 10(2):203–13. 10.1038/ni.1692 19122653

[161] Smeekens SP van de Veerdonk FL van der Meer JW Kullberg BJ Joosten LA Netea MG. The *Candida* Th17 response is dependent on mannan- and beta-glucan-induced prostaglandin E2. Int Immunol (2010) 22(11):889–95. 10.1093/intimm/dxq442 21059767

[162] van de Veerdonk FL Joosten LA Devesa I Mora-Montes HM Kanneganti TD Dinarello CA Bypassing pathogen-induced inflammasome activation for the regulation of interleukin-1beta production by the fungal pathogen *Candida albicans*. J Infect Dis (2009) 199(7):1087–96. 10.1086/597274 19222370

[163] Hise AG Tomalka J Ganesan S Patel K Hall BA Brown GD An essential role for the NLRP3 inflammasome in host defense against the human fungal pathogen *Candida albicans*. Cell Host Microbe (2009) 5(5):487–97. 10.1016/j.chom.2009.05.002 19454352 PMC2824856

[164] Gringhuis SI Kaptein TM Wevers BA Theelen B van der Vlist M Boekhout T Dectin-1 is an extracellular pathogen sensor for the induction and processing of IL-1 beta via a noncanonical caspase-8 inflammasome. Nat Immunol (2012) 13(3):246–54. 10.1038/ni.2222 22267217

[165] Ganesan S Rathinam VAK Bossaller L Army K Kaiser WJ Mocarski ES Caspase-8 modulates Dectin-1 and complement receptor 3-driven IL-1beta production in response to beta-glucans and the fungal pathogen, *Candida albicans*. J Immunol (2014) 193(5):2519–30. 10.4049/jimmunol.1400276 25063877 PMC4134963

[166] LeibundGut-Landmann S Gross O Robinson MJ Osorio F Slack EC Tsoni SV Syk- and CARD9-dependent coupling of innate immunity to the induction of T helper cells that produce interleukin 17. Nat Immunol (2007) 8(6):630–8. 10.1038/ni1460 17450144

[167] Takahara K Tokieda S Nagaoka K Takeda T Kimura Y Inaba K. C-type lectin SIGNR1 enhances cellular oxidative burst response against *C. albicans* in cooperation with Dectin-1. Eur J Immunol (2011) 41(5):1435–44. 10.1002/eji.200940188 21400494

[168] Li X Utomo A Cullere X Choi MM Milner DA Jr Venkatesh D The beta-glucan receptor Dectin-1 activates the integrin Mac-1 in neutrophils via Vav protein signaling to promote *Candida albicans* clearance. Cell Host Microbe (2011) 10(6):603–15. 10.1016/j.chom.2011.10.009 22177564 PMC3244687

[169] Le HT Tran VG Kim W Kim J Cho HR Kwon B. IL-33 priming regulates multiple steps of the neutrophil-mediated anti-*Candida albicans* response by modulating TLR and Dectin-1 signals. J Immunol (2012) 189(1):287–95. 10.4049/jimmunol.1103564 22661085

[170] Zawrotniak M Bochenska O Karkowska-Kuleta J Seweryn-Ozog K Aoki W Ueda M Aspartic proteases and major cell wall components in *Candida albicans* trigger the release of neutrophil extracellular traps. Front Cell Infect Microbiol (2017) 7:414. 10.3389/fcimb.2017.00414 28983472 PMC5613151

[171] Byrd AS O’Brien XM Johnson CM Lavigne LM Reichner JS. An extracellular matrix-based mechanism of rapid neutrophil extracellular trap formation in response to *Candida albicans*. J Immunol (2013) 190(8):4136–48. 10.4049/jimmunol.1202671 23509360 PMC3622194

[172] Blanco-Menendez N Del Fresno C Fernandes S Calvo E Conde-Garrosa R Kerr WG SHIP-1 Couples to the Dectin-1 hemITAM and selectively modulates reactive oxygen species production in dendritic cells in response to *Candida albicans*. J Immunol (2015) 195(9):4466–78. 10.4049/jimmunol.1402874 26416276 PMC4641325

[173] Rieber N Singh A Oz H Carevic M Bouzani M Amich J Pathogenic fungi regulate immunity by inducing neutrophilic myeloid-derived suppressor cells. Cell Host Microbe (2015) 17(4):507–14. 10.1016/j.chom.2015.02.007 25771792 PMC4400268

[174] Drummond RA Dambuza IM Vautier S Taylor JA Reid DM Bain CC CD4(+) T-cell survival in the GI tract requires Dectin-1 during fungal infection. Mucosal Immunol (2016) 9(2):492–502. 10.1038/mi.2015.79 26349660 PMC4677461

[175] Taylor PR Tsoni SV Willment JA Dennehy KM Rosas M Findon H Dectin-1 is required for beta-glucan recognition and control of fungal infection. Nat Immunol (2007) 8(1):31–8. 10.1038/ni1408 17159984 PMC1888731

[176] Saijo S Fujikado N Furuta T Chung SH Kotaki H Seki K Dectin-1 is required for host defense against *Pneumocystis carinii* but not against *Candida albicans*. Nat Immunol (2007) 8(1):39–46. 10.1038/ni1425 17159982

[177] Marakalala MJ Vautier S Potrykus J Walker LA Shepardson KM Hopke A Differential adaptation of *Candida albicans* in vivo modulates immune recognition by Dectin-1. PLoS Pathog (2013) 9(4):e1003315. 10.1371/journal.ppat.1003315 23637604 PMC3630191

[178] Chen SM Shen H Zhang T Huang X Liu XQ Guo SY Dectin-1 plays an important role in host defense against systemic *Candida glabrata* infection. Virulence (2017) 8:1643–56. 10.1080/21505594.2017.1346756 28658592 PMC5810470

[179] Ferwerda B Ferwerda G Plantinga TS Willment JA van Spriel AB Venselaar H Human Dectin-1 deficiency and mucocutaneous fungal infections. N Engl J Med (2009) 361(18):1760–7. 10.1056/NEJMoa0901053 19864674 PMC2773015

[180] Rosentul DC Plantinga TS Oosting M Scott WK Velez Edwards DR Smith PB Genetic variation in the Dectin-1/CARD9 recognition pathway and susceptibility to candidemia. J Infect Dis (2011) 204(7):1138–45. 10.1093/infdis/jir458 21881131 PMC3164426

[181] Plantinga TS van der Velden WJFM Ferwerda B van Spriel AB Adema G Feuth T Early stop polymorphism in human DECTIN-1 is associated with increased *Candida* colonization in hematopoietic stem cell transplant recipients. Clin Infect Dis (2009) 49(5):724–32. 10.1086/604714 19614557

[182] Plantinga TS Hamza OJM Willment JA Ferwerda B van de Geer NMD Verweij PE Genetic variation of innate immune genes in HIV-infected African patients with or without oropharyngeal candidiasis. J Acquir Immune Defic Syndr (2010) 55(1):87–94. 10.1097/QAI.0b013e3181e53c64 20577092 PMC3443739

[183] Robinson MJ Osorio F Rosas M Freitas RP Schweighoffer E Gross O Dectin-2 is a Syk-coupled pattern recognition receptor crucial for Th17 responses to fungal infection. J Exp Med (2009) 206(9):2037–51. 10.1084/jem.20082818 19703985 PMC2737172

[184] Bi L Gojestani S Wu W Hsu YM Zhu J Ariizumi K CARD9 mediates Dectin-2-induced IkappaBalpha kinase ubiquitination leading to activation of NF-kappaB in response to stimulation by the hyphal form of *Candida albicans*. J Biol Chem (2010) 285(34):25969–77. 10.1074/jbc.M110.131300 20538615 PMC2923990

[185] Ifrim DC Bain JM Reid DM Oosting M Verschueren I Gow NAR Role of Dectin-2 for host defense against systemic infection with *Candida glabrata*. Infect Immun (2014) 82(3):1064–73. 10.1128/IAI.01189-13 24343653 PMC3957982

[186] Ifrim DC Quintin J Courjol F Verschueren I van Krieken JH Koentgen F The role of Dectin-2 for host defense against disseminated candidiasis. J Interferon Cytokine Res (2016) 36(4):267–76. 10.1089/jir.2015.0040 27046240 PMC4827303

[187] Gorjestani S Yu M Tang B Zhang DK Wang DM Lin X Phospholipase C gamma 2 (PLC gamma 2) is key component in Dectin-2 signaling pathway, mediating anti-fungal innate immune responses. J Biol Chem (2011) 286(51):43651–9. 10.1074/jbc.M111.307389 22041900 PMC3243564

[188] Marodi L Korchak HM Johnston RB Mechanisms of host defense against *Candida* species. I. Phagocytosis by monocytes and monocyte-derived macrophages. J Immunol (1991) 146(8):2783–9. 1901885

[189] Yamamoto Y Klein TW Friedman H Involvement of mannose receptor in cytokine interleukin-1beta (IL-1beta), IL-6, and granulocyte-macrophage colony-stimulating factor responses, but not in chemokine macrophage inflammatory protein 1beta (MIP-1beta), MIP-2, and KC responses, caused by attachment of *Candida albicans* to macrophages. Infect Immun (1997) 65(3):1077–82. 9038318 10.1128/iai.65.3.1077-1082.1997 PMC175090

[190] Newman SL Holly A. *Candida albicans* is phagocytosed, killed, and processed for antigen presentation by human dendritic cells. Infect Immun (2001) 69(11):6813–22. 10.1128/IAI.69.11.6813-6822.2001 11598054 PMC100059

[191] Romani L Montagnoli C Bozza S Perruccio K Spreca A Allavena P The exploitation of distinct recognition receptors in dendritic cells determines the full range of host immune relationships with *Candida albicans*. Int Immunol (2004) 16(1):149–61. 10.1093/intimm/dxh012 14688070

[192] Claudia M Bacci A Silvia B Gaziano R Spreca A Romani L. The interaction of fungi with dendritic cells: implications for Th immunity and vaccination. Curr Mol Med (2002) 2(6):507–24. 10.2174/1566524023362203 12243244

[193] Gazi U Rosas M Singh S Heinsbroek S Haq I Johnson S Fungal recognition enhances mannose receptor shedding through Dectin-1 engagement. J Biol Chem (2011) 286(10):7822–9. 10.1074/jbc.M110.185025 21205820 PMC3048669

[194] Shepherd VL Lane KB Abdolrasulnia R. Ingestion of *Candida albicans* down-regulates mannose receptor expression on rat macrophages. Arch Biochem Biophys (1997) 344(2):350–6. 10.1006/abbi.1997.0219 9264549

[195] Heinsbroek SE Taylor PR Martinez FO Martinez-Pomares L Brown GD Gordon S. Stage-specific sampling by pattern recognition receptors during *Candida albicans* phagocytosis. PLoS Pathog (2008) 4(11):e1000218. 10.1371/journal.ppat.1000218 19043561 PMC2583056

[196] Lee SJ Zheng NY Clavijo M Nussenzweig MC. Normal host defense during systemic candidiasis in mannose receptor-deficient mice. Infect Immun (2003) 71(1):437–45. 10.1128/IAI.71.1.437-445.2003 12496194 PMC143203

[197] van de Veerdonk FL Marijnissen RJ Kullberg BJ Koenen HJ Cheng SC Joosten I The macrophage mannose receptor induces IL-17 in response to *Candida albicans*. Cell Host Microbe (2009) 5(4):329–40. 10.1016/j.chom.2009.02.006 19380112

[198] Smeekens SP van de Veerdonk FL Joosten LA Jacobs L Jansen T Williams DL The classical CD14(+)(+) CD16(-) monocytes, but not the patrolling CD14(+) CD16(+) monocytes, promote Th17 responses to *Candida albicans*. Eur J Immunol (2011) 41(10):2915–24. 10.1002/eji.201141418 21695694

[199] Castro M Ralston NVC Morgenthaler TI Rohrbach MS Limper AH *Candida albicans* stimulates arachidonic-acid liberation from alveolar macrophages through alpha-mannan and beta-glucan cell-wall components. Infect Immun (1994) 62(8):3138–45. 8039882 10.1128/iai.62.8.3138-3145.1994 PMC302938

[200] Estrada-Mata E Navarro-Arias MJ Perez-Garcia LA Mellado-Mojica E Lopez MG Csonka K Members of the *Candida parapsilosis* complex and *Candida albicans* are differentially recognized by human peripheral blood mononuclear cells. Front Microbiol (2016) 6:1527. 10.3389/fmicb.2015.01527 26793173 PMC4710749

[201] Donini M Zenaro E Tamassia N Dusi S. NADPH oxidase of human dendritic cells: role in *Candida albicans* killing and regulation by interferons, Dectin-1 and CD206. Eur J Immunol (2007) 37(5):1194–203. 10.1002/eji.200636532 17407098

[202] Marodi L Schreiber S Anderson DC Macdermott RP Korchak HM Johnston RB Enhancement of macrophage candidacidal activity by interferon-gamma – increased phagocytosis, killing, and calcium signal mediated by a decreased number of mannose receptors. J Clin Invest (1993) 91(6):2596–601. 10.1172/JCI116498 8390485 PMC443323

[203] Pellis V De Seta F Crovella S Bossi F Bulla R Guaschino S Mannose binding lectin and C3 act as recognition molecules for infectious agents in the vagina. Clin Exp Immunol (2005) 139(1):120–6. 10.1111/j.1365-2249.2005.02660.x 15606621 PMC1809267

[204] Ip WK Lau YL. Role of mannose-binding lectin in the innate defense against *Candida albicans*: enhancement of complement activation, but lack of opsonic function, in phagocytosis by human dendritic cells. J Infect Dis (2004) 190:632–40. 10.1086/422397 15243942

[205] Brouwer N Dolman KM van Houdt M Sta M Roos D Kuijpers TW. Mannose-binding lectin (MBL) facilitates opsonophagocytosis of yeasts but not of bacteria despite MBL binding. J Immunol (2008) 180(6):4124–32. 10.4049/jimmunol.180.6.4124 18322223

[206] van Asbeck EC Hoepelman AI Scharringa J Herpers BL Verhoef J. Mannose binding lectin plays a crucial role in innate immunity against yeast by enhanced complement activation and enhanced uptake of polymorphonuclear cells. BMC Microbiol (2008) 8:229. 10.1186/1471-2180-8-229 19094203 PMC2627907

[207] Li D Dong B Tong Z Wang Q Liu W Wang Y MBL-mediated opsonophagocytosis of *Candida albicans* by human neutrophils is coupled with intracellular Dectin-1-triggered ROS production. PLoS One (2012) 7:e50589. 10.1371/journal.pone.0050589 23239982 PMC3519760

[208] Held K Thiel S Loos M Petry F. Increased susceptibility of complement factor B/C2 double knockout mice and mannan-binding lectin knockout mice to systemic infection with *Candida albicans*. Mol Immunol (2008) 45(15):3934–41. 10.1016/j.molimm.2008.06.021 18672286

[209] Choteau L Parny M Francois N Bertin B Fumery M Dubuquoy L Role of mannose-binding lectin in intestinal homeostasis and fungal elimination. Mucosal Immunol (2016) 9(3):767–76. 10.1038/mi.2015.100 26442658

[210] Wang M Wang F Yang J Zhao D Wang H Shao F Mannan-binding lectin inhibits *Candida albicans*-induced cellular responses in PMA-activated THP-1 cells through toll-like receptor 2 and toll-like receptor 4. PLoS One (2013) 8:e83517. 10.1371/journal.pone.0083517 24391778 PMC3877063

[211] Liu C He T Rong Y Du F Ma D Wei Y Association of mannose-binding lectin polymorphisms with tuberculosis susceptibility among Chinese. Sci Rep (2016) 6:36488. 10.1038/srep36488 27812036 PMC5095599

[212] Toivonen L Vuononvirta J Mertsola J Waris M He Q Peltola V. Polymorphisms of mannose-binding lectin and toll-like receptors 2, 3, 4, 7 and 8 and the risk of respiratory infections and acute otitis media in children. Pediatr Infect Dis J (2017) 36(5):e114–22. 10.1097/INF.0000000000001479 28403045

[213] Babula O Lazdane G Kroica J Linhares IM Ledger WJ Witkin SS. Frequency of interleukin-4 (IL-4) -589 gene polymorphism and vaginal concentrations of IL-4, nitric oxide, and mannose-binding lectin in women with recurrent vulvovaginal candidiasis. Clin Infect Dis (2005) 40(9):1258–62. 10.1086/429246 15825027

[214] Liu F Liao Q Liu Z. Mannose-binding lectin and vulvovaginal candidiasis. Int J Gynaecol Obstet (2006) 92(1):43–7. 10.1016/j.ijgo.2005.08.024 16256117

[215] Babula O Lazdane G Kroica J Ledger WJ Witkin SS. Relation between recurrent vulvovaginal candidiasis, vaginal concentrations of mannose-binding lectin, and a mannose-binding lectin gene polymorphism in Latvian women. Clin Infect Dis (2003) 37(5):733–7. 10.1086/377234 12942410

[216] van Till JW Modderman PW de Boer M Hart MH Beld MG Boermeester MA. Mannose-binding lectin deficiency facilitates abdominal *Candida* infections in patients with secondary peritonitis. Clin Vaccine Immunol (2008) 15(1):65–70. 10.1128/CVI.00297-07 17978009 PMC2223851

[217] Osthoff M Wojtowicz A Tissot F Jorgensen C Thiel S Zimmerli S Association of lectin pathway proteins with intra-abdominal *Candida* infection in high-risk surgical intensive-care unit patients. A prospective cohort study within the fungal infection network of Switzerland. J Infect (2016) 72(3):377–85. 10.1016/j.jinf.2015.12.011 26730718

[218] Giraldo PC Babula O Goncalves AK Linhares IM Amaral RL Ledger WJ Mannose-binding lectin gene polymorphism, vulvovaginal candidiasis, and bacterial vaginosis. Obstet Gynecol (2007) 109(5):1123–8. 10.1097/01.AOG.0000260386.17555.a5 17470593

[219] Donders GG Babula O Bellen G Linhares IM Witkin SS. Mannose-binding lectin gene polymorphism and resistance to therapy in women with recurrent vulvovaginal candidiasis. BJOG (2008) 115(10):1225–31. 10.1111/j.1471-0528.2008.01830.x 18715406

[220] Nedovic B Posteraro B Leoncini E Ruggeri A Amore R Sanguinetti M Mannose-binding lectin codon 54 gene polymorphism and vulvovaginal candidiasis: a systematic review and meta-analysis. Biomed Res Int (2014) 2014:738298. 10.1155/2014/738298 25143944 PMC3910669

[221] Granell M Urbano-Ispizua A Suarez B Rovira M Fernandez-Aviles F Martinez C Mannan-binding lectin pathway deficiencies and invasive fungal infections following allogeneic stem cell transplantation. Exp Hematol (2006) 34(10):1435–41. 10.1016/j.exphem.2006.06.005 16982337

[222] de Mare-Bredemeijer EL Mancham S Utomo WK de Canck I van Thielen M de Meester E Genetic polymorphisms in innate immunity receptors do not predict the risk of bacterial and fungal infections and acute rejection after liver transplantation. Transpl Infect Dis (2013) 15(2):120–33. 10.1111/tid.12034 23240652

[223] Beltrame MH Boldt AB Catarino SJ Mendes HC Boschmann SE Goeldner I MBL-associated serine proteases (MASPs) and infectious diseases. Mol Immunol (2015) 67(1):85–100. 10.1016/j.molimm.2015.03.245 25862418 PMC7112674

[224] Klassert TE Goyal S Stock M Driesch D Hussain A Berrocal-Almanza LC AmpliSeq screening of genes encoding the C-type lectin receptors and their signaling components reveals a common variant in MASP1 associated with pulmonary tuberculosis in an Indian population. Front Immunol (2018) 9:242. 10.3389/fimmu.2018.00242 29515573 PMC5826192

[225] te Riet J Reinieren-Beeren I Figdor CG Cambi A AFM force spectroscopy reveals how subtle structural differences affect the interaction strength between *Candida albicans* and DC-SIGN. J Mol Recognit (2015) 28(11):687–98. 10.1002/jmr.2481 26011000

[226] Netea MG Gijzen K Coolen N Verschueren I Figdor C Van der Meer JWM Human dendritic cells are less potent at killing *Candida albicans* than both monocytes and macrophages. Microbes Infect (2004) 6(11):985–9. 10.1016/j.micinf.2004.05.013 15345229

[227] Mittal R Bulgheresi S Emami C Prasadarao NV. *Enterobacter sakazakii* targets DC-SIGN to induce immunosuppressive responses in dendritic cells by modulating MAPKs. J Immunol (2009) 183(10):6588–99. 10.4049/jimmunol.0902029 19846880 PMC2796599

[228] Gringhuis SI den Dunnen J Litjens M van Het Hof B van Kooyk Y Geijtenbeek TB. C-type lectin DC-SIGN modulates toll-like receptor signaling via Raf-1 kinase-dependent acetylation of transcription factor NF-kappaB. Immunity (2007) 26(5):605–16. 10.1016/j.immuni.2007.03.012 17462920

[229] Takahara K Tokieda S Nagaoka K Inaba K Efficient capture of *Candida albicans* and zymosan by SIGNR1 augments TLR2-dependent TNF-alpha production. Int Immunol (2012) 24(2):89–96. 10.1093/intimm/dxr103 22207132

[230] Bugarcic A Hitchens K Beckhouse AG Wells CA Ashman RB Blanchard H. Human and mouse macrophage-inducible C-type lectin (Mincle) bind *Candida albicans*. Glycobiology (2008) 18(9):679–85. 10.1093/glycob/cwn046 18509109

[231] Vijayan D Radford KJ Beckhouse AG Ashman RB Wells CA. Mincle polarizes human monocyte and neutrophil responses to *Candida albicans*. Immunol Cell Biol (2012) 90(9):889–95. 10.1038/icb.2012.24 22641025

[232] Szabo I Guan LM Rogers TJ Modulation of macrophage phagocytic-activity by cell-wall components of *Candida albicans*. Cell Immunol (1995) 164(2):182–8. 10.1006/cimm.1995.1160 7656326

[233] Forsyth CB Mathews HL. Lymphocyte adhesion to *Candida albicans*. Infect Immun (2002) 70(2):517–27. 10.1128/IAI.70.2.517-527.2002 11796578 PMC127679

[234] Forsyth CB Mathews HL. Lymphocytes utilize CD11b/CD18 for adhesion to *Candida albicans*. Cell Immunol (1996) 170(1):91–100. 10.1006/cimm.1996.0138 8660804

[235] Losse J Svobodova E Heyken A Hube B Zipfel PF Jozsi M. Role of pH-regulated antigen 1 of *Candida albicans* in the fungal recognition and antifungal response of human neutrophils. Mol Immunol (2011) 48(15–16):2135–43. 10.1016/j.molimm.2011.07.007 21820180

[236] Choi W Yoo YJ Kim M Shin D Jeon HB Choi W. Identification of proteins highly expressed in the hyphae of *Candida albicans* by two-dimensional electrophoresis. Yeast (2003) 20(12):1053–60. 10.1002/yea.1022 12961753

[237] Soloviev DA Jawhara S Fonzi WA Regulation of innate immune response to *Candida albicans* infections by alpha(M)beta(2)-Pra1p interaction. Infect Immun (2011) 79(4):1546–58. 10.1128/IAI.00650-10 21245270 PMC3067562

[238] Thornton BP Vetvicka V Pitman M Goldman RC Ross GD. Analysis of the sugar specificity and molecular location of the beta-glucan-binding lectin site of complement receptor type 3 (CD11b/CD18). J Immunol (1996) 156(3):1235–46. 8558003

[239] Le Cabec V Carreno S Moisand A Bordier C Maridonneau-Parini I. Complement receptor 3 (CD11b/CD18) mediates type I and type II phagocytosis during nonopsonic and opsonic phagocytosis, respectively. J Immunol (2002) 169(4):2003–9. 10.4049/jimmunol.169.4.2003 12165526

[240] Lavigne LM Albina JE Reichner JS. Beta-glucan is a fungal determinant for adhesion-dependent human neutrophil functions. J Immunol (2006) 177(12):8667–75. 10.4049/jimmunol.177.12.8667 17142767

[241] Tateno H Ohnishi K Yabe R Hayatsu N Sato T Takeya M Dual specificity of Langerin to sulfated and mannosylated glycans via a single C-type carbohydrate recognition domain. J Biol Chem (2010) 285(9):6390–400. 10.1074/jbc.M109.041863 20026605 PMC2825434

[242] De Jesus M Rodriguez AE Yagita H Ostroff GR Mantis NJ. Sampling of *Candida albicans* and *Candida tropicalis* by langerin-positive dendritic cells in mouse Peyer’s patches. Immunol Lett (2015) 168(1):64–72. 10.1016/j.imlet.2015.09.008 26386376

[243] Igyarto BZ Haley K Ortner D Bobr A Gerami-Nejad M Edelson BT Skin-resident murine dendritic cell subsets promote distinct and opposing antigen-specific T helper cell responses. Immunity (2011) 35(2):260–72. 10.1016/j.immuni.2011.06.005 21782478 PMC3163010

[244] Haley K Igyarto BZ Ortner D Bohr A Kashem S Schenten D Langerhans cells require MyD88-dependent signals for *Candida albicans* response but not for contact hypersensitivity or migration. J Immunol (2012) 188(9):4334–9. 10.4049/jimmunol.1102759 22442445 PMC3331889

[245] Illnait-Zaragozi MT Martínez-Machín GF Fernández-Andreu CM Perurena-Lancha MR Hagen F Meis JF *Cryptococcus* and cryptococcosis in Cuba. A minireview. Mycoses (2014) 57:707–17. 10.1111/myc.12275 25420448

[246] May RC Stone NRH Wiesner DL Bicanic T Nielsen K *Cryptococcus*: from environmental saprophyte to global pathogen. Nat Rev Microbiol (2015) 14:106–17. 10.1038/nrmicro.2015.6 26685750 PMC5019959

[247] Cross CE Bancroft GJ. Ingestion of acapsular *Cryptococcus neoformans* occurs via mannose and beta-glucan receptors, resulting in cytokine production and increased phagocytosis of the encapsulated form. Infect Immun (1995) 63:2604–11. 7790075 10.1128/iai.63.7.2604-2611.1995 PMC173349

[248] Nakamura K Kinjo T Saijo S Miyazato A Adachi Y Ohno N Dectin-1 is not required for the host defense to *Cryptococcus neoformans*. Microbiol Immunol (2007) 51:1115–9. 10.1111/j.1348-0421.2007.tb04007.x 18037789

[249] Nakamura Y Sato K Yamamoto H Matsumura K Matsumoto I Nomura T Dectin-2 deficiency promotes Th2 response and mucin production in the lungs after pulmonary infection with *Cryptococcus neoformans*. Infect Immun (2015) 83:671–81. 10.1128/IAI.02835-14 25422263 PMC4294247

[250] Dan JM Kelly RM Lee CK Levitz SM. Role of the mannose receptor in a murine model of *Cryptococcus neoformans* infection. Infect Immun (2008) 76:2362–7. 10.1128/IAI.00095-08 18391001 PMC2423054

[251] Syme RM Spurrell JCL Amankwah EK Green FHY Mody CH. Primary dendritic cells phagocytose *Cryptococcus neoformans* via mannose receptors and Fcgamma receptor II for presentation to T lymphocytes. Infect Immun (2002) 70:5972–81. 10.1128/IAI.70.11.5972-5981.2002 12379672 PMC130340

[252] Walenkamp AM Verheul AF Scharringa J Hoepelman IM. Pulmonary surfactant protein A binds to *Cryptococcus neoformans* without promoting phagocytosis. Eur J Clin Invest (1999) 29:83–92. 10.1046/j.1365-2362.1999.00429.x 10092994

[253] Geunes-Boyer S Beers MF Perfect JR Heitman J Wright JR. Surfactant protein D facilitates *Cryptococcus neoformans* infection. Infect Immun (2012) 80:2444–53. 10.1128/IAI.05613-11 22547543 PMC3416458

[254] Giles SS Zaas AK Reidy MF Perfect JR Wright JR. *Cryptococcus neoformans* is resistant to surfactant protein A mediated host defense mechanisms. PLoS One (2007) 2:e1370. 10.1371/journal.pone.0001370 18159253 PMC2147053

[255] Holmer SM Evans KS Asfaw YG Saini D Schell WA Ledford JG Impact of surfactant protein D, interleukin-5, and eosinophilia on cryptococcosis. Infect Immun (2014) 82:683–93. 10.1128/IAI.00855-13 24478083 PMC3911392

[256] Geunes-Boyer S Oliver TN Janbon G Lodge JK Heitman J Perfect JR Surfactant protein D increases phagocytosis of hypocapsular *Cryptococcus neoformans* by murine macrophages and enhances fungal survival. Infect Immun (2009) 77:2783–94. 10.1128/IAI.00088-09 19451250 PMC2708589

[257] Panepinto JC Komperda KW Hacham M Shin S Liu X Williamson PR Binding of serum mannan binding lectin to a cell integrity-defective *Cryptococcus neoformans* ccr4 mutant. Infect Immun (2007) 75:4769–79. 10.1128/IAI.00536-07 17646356 PMC2044520

[258] Ibrahim AS Spellberg B Walsh TJ Kontoyiannis DP. Pathogenesis of mucormycosis. Clin Infect Dis (2012) 54:S16–22. 10.1093/cid/cir865 22247441 PMC3286196

[259] Katragkou A Walsh TJ Roilides E. Why is mucormycosis more difficult to cure than more common mycoses? Clin Microbiol Infect (2014) 20:74–81. 10.1111/1469-0691.12466 24279587

[260] Mendoza L Vilela R Voelz K Ibrahim AS Voigt K Lee SC Human fungal pathogens of mucorales and entomophthorales. Cold Spring Harb Perspect Med (2015) 5(4). 10.1101/cshperspect.a019562 PMC4382724 25377138

[261] Gigliotti F Limper AH Wright T. *Pneumocystis*. Cold Spring Harb Perspect Med (2014) 4:a019828. 10.1101/cshperspect.a019828 25367973 PMC4292088

[262] Vassallo R Standing JE Limper AH Isolated *Pneumocystis carinii* cell wall glucan provokes lower respiratory tract inflammatory responses. J Immunol (2000) 164:3755–63. 10.4049/jimmunol.164.7.3755 10725735

[263] Hoffman OA Standing JE Limper AH *Pneumocystis carinii* stimulates tumor necrosis factor-alpha release from alveolar macrophages through a beta-glucan-mediated mechanism. J Immunol (1993) 150:3932–40. 8386203

[264] Nandakumar V Hebrink D Jenson P Kottom T Limper AH. Differential macrophage polarization from *Pneumocystis* in immunocompetent and immunosuppressed hosts: potential adjunctive therapy during pneumonia. Infect Immun (2017) 85:e939–916. 10.1128/IAI.00939-16 27993972 PMC5328482

[265] Zhang C Wang S-H Liao C-P Shao S Lasbury ME Durant PJ Downregulation of PU.1 leads to decreased expression of Dectin-1 in alveolar macrophages during *Pneumocystis* pneumonia. Infect Immun (2010) 78:1058–65. 10.1128/IAI.01141-09 20065023 PMC2825957

[266] Steele C Marrero L Swain S Harmsen AG Zheng M Brown GD Alveolar macrophage-mediated killing of *Pneumocystis carinii* f. sp. muris involves molecular recognition by the Dectin-1 β-glucan receptor. J Exp Med (2003) 198:1677–88. 10.1084/jem.20030932 14657220 PMC2194130

[267] Evans SE Hahn PY McCann F Kottom TJ Pavlovic’ ZV Limper AH *Pneumocystis* cell wall β-glucans stimulate alveolar epithelial cell chemokine generation through nuclear factor-κB-dependent mechanisms. Am J Respir Cell Mol Biol (2005) 32:490–7. 10.1165/rcmb.2004-0300OC 15746433 PMC2715319

[268] Hahn PY Evans SE Kottom TJ Standing JE Pagano RE Limper AH *Pneumocystis carinii* cell wall β-glucan induces release of macrophage inflammatory protein-2 from alveolar epithelial cells via a lactosylceramide-mediated mechanism. J Biol Chem (2003) 278:2043–50. 10.1074/jbc.M209715200 12419803

[269] Fraser IP Takahashi K Koziel H Fardin B Harmsen A Ezekowitz RAB. *Pneumocystis carinii* enhances soluble mannose receptor production by macrophages. Microbes Infect (2000) 2:1305–10. 10.1016/S1286-4579(00)01283-1 11018446

[270] Zhang J Zhu J Bu X Cushion M Kinane TB Avraham H Cdc42 and RhoB activation are required for mannose receptor-mediated phagocytosis by human alveolar macrophages. Mol Biol Cell (2005) 16:824–34. 10.1091/mbc.e04-06-0463 15574879 PMC545914

[271] Stehle SE Rogers RA Harmsen AG Ezekowitz RA. A soluble mannose receptor immunoadhesin enhances phagocytosis of *Pneumocystis carinii* by human polymorphonuclear leukocytes in vitro. Scand J Immunol (2000) 52:131–7. 10.1046/j.1365-3083.2000.00755.x 10931380

[272] Ezekowitz RAB Williams DJ Koziel H Armstrong MYK Warner A Richards FF Uptake of *Pneumocystis carinii* mediated by the macrophage mannose receptor. Nature (1991) 351:155–8. 10.1038/351155a0 1903183

[273] Koziel H Eichbaum Q Kruskal BA Pinkston P Rogers RA Armstrong MY Reduced binding and phagocytosis of *Pneumocystis carinii* by alveolar macrophages from persons infected with HIV-1 correlates with mannose receptor downregulation. J Clin Invest (1998) 102:1332–44. 10.1172/JCI560 9769325 PMC508980

[274] Tachado SD Zhang J Zhu J Patel N Cushion M Koziel H *Pneumocystis*-mediated IL-8 release by macrophages requires coexpression of mannose receptors and TLR2. J Leukoc Biol (2006) 81:205–11. 10.1189/jlb.1005580 17020928

[275] Zhang J Tachado SD Patel N Zhu J Imrich A Manfruelli P Negative regulatory role of mannose receptors on human alveolar macrophage proinflammatory cytokine release in vitro. J Leukoc Biol (2005) 78:665–74. 10.1189/jlb.1204699 16000387

[276] Linke M Ashbaugh A Koch J Tanaka R Walzer P. Surfactant protein A limits *Pneumocystis murina* infection in immunosuppressed C3H/HeN mice and modulates host response during infection. Microbes Infect (2005) 7:748–59. 10.1016/j.micinf.2005.01.011 15857803

[277] Atochina EN Beck JM Preston AM Haczku A Tomer Y Scanlon ST Enhanced lung injury and delayed clearance of *Pneumocystis carinii* in surfactant protein A-deficient mice: attenuation of cytokine responses and reactive oxygen-nitrogen species. Infect Immun (2004) 72:6002–11. 10.1128/IAI.72.10.6002-6011.2004 15385504 PMC517574

[278] Atochina Elena N Gow Andrew J Beck James M Haczku A Inch A Kadire H Delayed clearance of *Pneumocystis carinii* infection, increased inflammation, and altered nitric oxide metabolism in lungs of surfactant protein-D knockout mice. J Infect Dis (2004) 189:1528–39. 10.1086/383130 15073692

[279] Linke MJ Ashbaugh AD Demland JA Walzer PD. *Pneumocystis murina* colonization in immunocompetent surfactant protein A deficient mice following environmental exposure. Respir Res (2009) 10:10. 10.1186/1465-9921-10-10 19228388 PMC2650685

[280] Linke M Ashbaugh A Koch J Tanaka R Walzer P. Efficient resolution of *Pneumocystis murina* infection in surfactant protein A-deficient mice following withdrawal of corticosteroid-induced immunosuppression. J Med Microbiol (2006) 55:143–7. 10.1099/jmm.0.46190-0 16434705

[281] Linke Michael J Harris Christopher E Korfhagen Thomas R McCormack Francis X Ashbaugh Alan D Steele P Immunosuppressed surfactant protein A-deficient mice have increased susceptibility to *Pneumocystis carinii* infection. J Infect Dis (2001) 183:943–52. 10.1086/319252 11237812

[282] Zhu S Kachel DL Martin WJ Matalon S Nitrated SP-A does not enhance adherence of *Pneumocystis carinii* to alveolar macrophages. Am J Physiol (1998) 275:L1031–9. 9843839 10.1152/ajplung.1998.275.6.L1031

[283] Williams MD Wright JR March KL Martin WJ. Human surfactant protein A enhances attachment of *Pneumocystis carinii* to rat alveolar macrophages. Am J Respir Cell Mol Biol (1996) 14:232–8. 10.1165/ajrcmb.14.3.8845173 8845173

[284] Limper AH Crouch EC O’Riordan DM Chang D Vuk-Pavlovic Z Standing JE Surfactant protein-D modulates interaction of *Pneumocystis carinii* with alveolar macrophages. J Lab Clin Med (1995) 126:416–22. 7595025

[285] Koziel H Phelps DS Fishman JA Armstrong MYK Richards FF Rose RM Surfactant protein-A reduces binding and phagocytosis of *Pneumocystis carinii* by human alveolar macrophages in vitro. Am J Respir Cell Mol Biol (1998) 18:834–43. 10.1165/ajrcmb.18.6.3059 9618388

[286] Yong S-J Vuk-Pavlovic Z Standing JE Crouch EC Limper AH. Surfactant protein D-mediated aggregation of *Pneumocystis carinii* impairs phagocytosis by alveolar macrophages. Infect Immun (2003) 71:1662–71. 10.1128/IAI.71.4.1662-1671.2003 12654779 PMC152070

[287] Vuk-Pavlovic Z Mo EK Icenhour CR Standing JE Fisher JH Limper AH Surfactant protein D enhances *Pneumocystis* infection in immune-suppressed mice. Am J Physiol Lung Cell Mol Physiol (2005) 290:L442–9. 10.1152/ajplung.00112.2005 16199436

[288] Linke MJ Ashbaugh AA Koch JV Levin L Tanaka R Walzer PD Effects of surfactant protein-A on the interaction of *Pneumocystis murina* with its host at different stages of the infection in mice. J Eukaryot Microbiol (2009) 56:58–65. 10.1111/j.1550-7408.2008.00363.x 19335775 PMC2675919

[289] O’Riordan DM Standing JE Kwon KY Chang D Crouch EC Limper AH. Surfactant protein D interacts with *Pneumocystis carinii* and mediates organism adherence to alveolar macrophages. J Clin Invest (1995) 95:2699–710. 10.1172/JCI117972 7769109 PMC295953

[290] Zimmerman PE Voelker DR McCormack FX Paulsrud JR Martin WJ. 120-kD surface glycoprotein of *Pneumocystis carinii* is a ligand for surfactant protein A. J Clin Invest (1992) 89:143–9. 10.1172/JCI115554 1530850 PMC442829

[291] Yanagisawa K Ogawa Y Uchiumi H Gohda F Mawatari M Ishizaki T Gene polymorphisms of mannose-binding lectin confer susceptibility to *Pneumocystis* pneumonia in HIV-infected patients. J Infect Chemother (2015) 21:769–75. 10.1016/j.jiac.2015.07.006 26271591

[292] Schildgen V Mai S Khalfaoui S Lüsebrink J Pieper M Tillmann RL *Pneumocystis jirovecii* can be productively cultured in differentiated CuFi-8 airway cells. MBio (2014) 5:e1186–14. 10.1128/mBio.01186-14 24825015 PMC4030487

[293] Skalski JH Kottom TJ Limper AH Pathobiology of *Pneumocystis* pneumonia: life cycle, cell wall and cell signal transduction. FEMS Yeast Res (2015) 15:fov046 10.1093/femsyr/fov046 26071598

[294] Powers-Fletcher MV Kendall BA Griffin AT Hanson KE Filamentous Fungi. Microbiol Spectr (2016) 4(3). 10.1128/microbiolspec.DMIH2-0002-2015 27337469

[295] del Pilar Jiménez AM Viriyakosol S Walls L Datta SK Kirkland T Heinsbroek SEM Susceptibility to *Coccidioides* species in C57BL/6 mice is associated with expression of a truncated splice variant of Dectin-1 (Clec7a). Genes Immun (2008) 9:338–48. 10.1038/gene.2008.23 18418396 PMC3681288

[296] Viriyakosol S Jimenez MDP Gurney MA Ashbaugh ME Fierer J Dectin-1 is required for resistance to coccidioidomycosis in mice. MBio (2013) 4:e597–12. 10.1128/mBio.00597-12 PMC3562125 23386437

[297] Ampel NM Dionne SO Giblin A Podany AB Galgiani J. Mannose-binding lectin serum levels are low in persons with clinically active coccidioidomycosis. Mycopathologia (2009) 167:173–80. 10.1007/s11046-008-9172-6 19083122

[298] Viriyakosol S Jimenez MDP Saijo S Fierer J. Neither Dectin-2 nor the mannose receptor is required for resistance to *Coccidioides* immitis in mice. Infect Immun (2014) 82:1147–56. 10.1128/IAI.01355-13 24379281 PMC3957980

[299] Garfoot AL Rappleye CA. *Histoplasma capsulatum* surmounts obstacles to intracellular pathogenesis. FEBS J (2016) 283:619–33. 10.1111/febs.13389 26235362 PMC4827932

[300] Nanjappa SG Heninger E Wüthrich M Gasper DJ Klein BS. Tc17 cells mediate vaccine immunity against lethal fungal pneumonia in immune deficient hosts lacking CD4+ T cells. PLoS Pathog (2012) 8:e1002771. 10.1371/journal.ppat.1002771 22829762 PMC3400565

[301] Garfoot AL Shen Q Wüthrich M Klein BS Rappleye CA The Eng1 β-glucanase enhances *Histoplasma* virulence by reducing β-glucan exposure. MBio (2016) 7:e1388–1315. 10.1128/mBio.01388-15 PMC4850272 27094334

[302] Rappleye CA Eissenberg LG Goldman WE. *Histoplasma capsulatum* alpha-(1,3)-glucan blocks innate immune recognition by the beta-glucan receptor. Proc Natl Acad Sci U S A (2007) 104:1366–70. 10.1073/pnas.0609848104 17227865 PMC1783108

[303] Gonzalez A Hernandez O New insights into a complex fungal pathogen: the case of *Paracoccidioides spp*. Yeast (2016) 33:113–28. 10.1002/yea.3147 26683539

[304] Rodrigues DR Fernandes RK Balderramas HDA Penitenti M Bachiega TF Calvi SA Interferon-gamma production by human neutrophils upon stimulation by IL-12, IL-15 and IL-18 and challenge with *Paracoccidioides brasiliensis*. Cytokine (2014) 69:102–9. 10.1016/j.cyto.2014.05.009 25022968

[305] Balderramas HA Penitenti M Rodrigues DR Bachiega TF Fernandes RK Ikoma MRVR Human neutrophils produce IL-12, IL-10, PGE2 and LTB4 in response to *Paracoccidioides brasiliensis*. Involvement of TLR2, mannose receptor and Dectin-1. Cytokine (2014) 67:36–43. 10.1016/j.cyto.2014.02.004 24680480

[306] Bachiega TF Dias-Melicio LA Fernandes RK de Almeida Balderramas H Rodrigues DR Ximenes VF Participation of Dectin-1 receptor on NETs release against *Paracoccidioides brasiliensis*: role on extracellular killing. Immunobiology (2016) 221:228–35. 10.1016/j.imbio.2015.09.003 26416210

[307] Loures FVV Araújo EF Feriotti C Bazan SB Costa TNA Brown GD Dectin-1 induces M1 macrophages and prominent expansion of CD8+IL-17+ cells in pulmonary paracoccidioidomycosis. J Infect Dis (2014) 210:762–73. 10.1093/infdis/jiu136 24604821

[308] Feriotti C Loures FV Frank de Araújo E Costa TAD Calich VLG. Mannosyl-recognizing receptors induce an M1-like phenotype in macrophages of susceptible mice but an M2-like phenotype in mice resistant to a fungal infection. PLoS One (2013) 8:e54845. 10.1371/journal.pone.0054845 23382985 PMC3559829

[309] Toledo RG Da Silva WD Calich VLG Kipnis TL. Mannose-binding lectin complement pathway plays a key role in complement activation by *Paracoccidioides brasiliensis*. Mol Immunol (2010) 48:26–36. 10.1016/j.molimm.2010.09.015 21035191

[310] Chan JFW Lau SKP Yuen KY Woo PCY *Talaromyces (Penicillium) marneffei* infection in non-HIV-infected patients. Emerg Microbes Infect (2016) 5:e19 10.1038/emi.2016.18 26956447 PMC4820671

[311] Vanittanakom N Cooper CR Jr Fisher MC Sirisanthana T. *Penicillium marneffei* infection and recent advances in the epidemiology and molecular biology aspects. Clin Microbiol Rev (2006) 19(1):95–110. 10.1128/CMR.19.1.95-110.2006 16418525 PMC1360277

[312] Zhan P Liu W. The changing face of dermatophytic infections worldwide. Mycopathologia (2017) 182(1–2):77–86. 10.1007/s11046-016-0082-8 27783316

[313] Yoshikawa FS Yabe R Iwakura Y de Almeida SR Saijo S Dectin-1 and Dectin-2 promote control of the fungal pathogen *Trichophyton rubrum* independently of IL-17 and adaptive immunity in experimental deep dermatophytosis. Innate Immun (2016) 22:316–24. 10.1177/1753425916645392 27189427

[314] Nakamura T Nishibu A Yasoshima M Tanoue C Yoshida N Hatta J Analysis of *Trichophyton* antigen-induced contact hypersensitivity in mouse. J Dermatol Sci (2012) 66:144–53. 10.1016/j.jdermsci.2012.02.008 22459756

[315] Velegraki A Cafarchia C Gaitanis G Iatta R Boekhout T Otranto D *Malassezia* infections in humans and animals: pathophysiology, detection, and treatment. PLoS Pathog (2015) 11:e1004523 10.1371/journal.ppat.1004523 25569140 PMC4287564

[316] Tragiannidis A Bisping G Koehler G Groll AH Minireview: *Malassezia* infections in immunocompromised patients. Mycoses (2010) 53:187–95. 10.1111/j.1439-0507.2009.01814.x 20028460

[317] Yamasaki S Matsumoto M Takeuchi O Matsuzawa T Ishikawa E Sakuma M C-type lectin Mincle is an activating receptor for pathogenic fungus, *Malassezia*. Proc Natl Acad Sci U S A (2009) 106:1897–902. 10.1073/pnas.0805177106 19171887 PMC2644135

[318] Van Der Vlist M Geijtenbeek TB. Langerin functions as an antiviral receptor on Langerhans cells. Immunol Cell Biol (2010) 88(4):410–5. 10.1038/icb.2010.32 20309013

[319] Santos ALS Palmeira VF Rozental S Kneipp LF Nimrichter L Alviano DS Biology and pathogenesis of *Fonsecaea pedrosoi*, the major etiologic agent of chromoblastomycosis. FEMS Microbiol Rev (2007) 31:570–91. 10.1111/j.1574-6976.2007.00077.x 17645522

[320] Wevers BA Kaptein TM Zijlstra-Willems EM Theelen B Boekhout T Geijtenbeek TBH Fungal engagement of the C-type lectin Mincle suppresses Dectin-1-induced antifungal immunity. Cell Host Microbe (2014) 15:494–505. 10.1016/j.chom.2014.03.008 24721577

[321] Skerlev M Miklic P. The changing face of *Microsporum* spp. infections. Clin Dermatol (2010) 28(2):146–50. 10.1016/j.clindermatol.2009.12.007 20347656

[322] Zahur M Afroz A Rashid U Khaliq S. Dermatomycoses: challenges and human immune responses. Curr Protein Pept Sci (2014) 15(5):437–44. 10.2174/1389203715666140512121349 24818759

[323] Mao LM Zhang LP Li H Chen W Wang HB Wu SX Pathogenic fungus *Microsporum canis* activates the NLRP3 inflammasome. Infect Immun (2014) 82(2):882–92. 10.1128/IAI.01097-13 24478101 PMC3911390

[324] Nucci M Anaissie E. *Fusarium* infections in immunocompromised patients. Clin Microbiol Rev (2007) 20(4):695–704. 10.1128/CMR.00014-07 17934079 PMC2176050

[325] Karthikeyan RS Leal SM Jr Prajna NV Dharmalingam K Geiser DM Pearlman E Expression of innate and adaptive immune mediators in human corneal tissue infected with *Aspergillus* or *Fusarium*. J Infect Dis (2011) 204(6):942–50. 10.1093/infdis/jir426 21828275 PMC3156922

[326] Kolar SS Baidouri H McDermott AM. Role of pattern recognition receptors in the modulation of antimicrobial peptide expression in the corneal epithelial innate response to *F. solani*. Invest Ophthalmol Vis Sci (2017) 58(5):2463–72. 10.1167/iovs.16-20658 28460048 PMC5413214

[327] Che CY Li XJ Jia WY Li N Xu Q Lin J Early expression of surfactant proteins D in *Fusarium solani* infected rat cornea. Int J Ophthalmol (2012) 5(3):297–300. 10.3980/j.issn.2222-3959.2012.03.09 22773976 PMC3388396

[328] Colombo AL Padovan ACB Chaves GM Current knowledge of *Trichosporon* spp. and trichosporonosis. Clin Microbiol Rev (2011) 24(4):682–700. 10.1128/CMR.00003-11 21976604 PMC3194827

[329] Almeida JND Hennequin C Invasive *Trichosporon* infection: a systematic review on a re-emerging fungal pathogen. Front Microbiol (2016) 7:1629 10.3389/fmicb.2016.01629 27799926 PMC5065970

[330] Duarte-Oliveira C Rodrigues F Goncalves SM Goldman GH Carvalho A Cunha C The cell biology of the *Trichosporon*-host interaction. Front Cell Infect Microbiol (2017) 7:118 10.3389/fcimb.2017.00118 28439501 PMC5383668

[331] Pérez-Torrado R Querol A Opportunistic strains of *Saccharomyces cerevisiae*: a potential risk sold in food products. Front Microbiol (2015) 6:1522 10.3389/fmicb.2015.01522 26779173 PMC4705302

[332] Anoop V Rotaru S Shwed PS Tayabali AF Arvanitakis G Review of current methods for characterizing virulence and pathogenicity potential of industrial *Saccharomyces cerevisiae* strains towards humans. FEMS Yeast Res (2015) 15:fov057 10.1093/femsyr/fov057 26195617

[333] Aucott JN Fayen J Grossnicklas H Morrissey A Lederman MM Salata RA. Invasive infection with *Saccharomyces cerevisiae*: report of three cases and review. Rev Infect Dis (1990) 12:406–11. 10.1093/clinids/12.3.406 2193348

[334] Rizzetto L Buschow SI Beltrame L Figdor CG Schierer S Schuler G The modular nature of dendritic cell responses to commensal and pathogenic fungi. PLoS One (2012) 7:e42430. 10.1371/journal.pone.0042430 22879980 PMC3411757

[335] Aimanianda V Clavaud C Simenel C Fontaine T Delepierre M Latgé J-P. Cell wall beta-(1,6)-glucan of *Saccharomyces cerevisiae*: structural characterization and in situ synthesis. J Biol Chem (2009) 284:13401–12. 10.1074/jbc.M807667200 19279004 PMC2679440

[336] Huang H Ostroff GR Lee CK Wang JP Specht CA Levitz SM. Distinct patterns of dendritic cell cytokine release stimulated by fungal beta-glucans and toll-like receptor agonists. Infect Immun (2009) 77:1774–81. 10.1128/IAI.00086-09 19273561 PMC2681737

[337] Roy S Dickerson R Khanna S Collard E Gnyawali U Gordillo GM Particulate β-glucan induces TNF-α production in wound macrophages via a redox-sensitive NF-κβ-dependent pathway. Wound Repair Regen (2011) 19:411–9. 10.1111/j.1524-475X.2011.00688.x 21518092 PMC3399431

[338] Yang Z Marshall JS. Zymosan treatment of mouse mast cells enhances Dectin-1 expression and induces Dectin-1-dependent reactive oxygen species (ROS) generation. Immunobiology (2009) 214:321–30. 10.1016/j.imbio.2008.09.002 19327548

[339] Karumuthil-Melethil S Gudi R Johnson BM Perez N Vasu C. Fungal β-glucan, a Dectin-1 ligand, promotes protection from type 1 diabetes by inducing regulatory innate immune response. J Immunol (2014) 193:3308–21. 10.4049/jimmunol.1400186 25143443 PMC4170060

[340] Qi C Cai Y Gunn L Ding C Li B Kloecker G Differential pathways regulating innate and adaptive antitumor immune responses by particulate and soluble yeast-derived β-glucans. Blood (2011) 117:6825–36. 10.1182/blood-2011-02-339812 21531981 PMC3128477

[341] Saegusa S Totsuka M Kaminogawa S Hosoi T *Saccharomyces cerevisiae* and *Candida albicans* stimulate cytokine secretion from human neutrophil-like HL-60 cells differentiated with retinoic acid or dimethylsulfoxide. Biosci Biotechnol Biochem (2009) 73:2600–8. 10.1271/bbb.90410 19966493

[342] Tam JM Mansour MK Khan NS Yoder NC Vyas JM. Use of fungal derived polysaccharide-conjugated particles to probe Dectin-1 responses in innate immunity. Integr Biol (Camb) (2012) 4:220–7. 10.1039/c2ib00089j 22200052 PMC3346694

[343] McCann F Carmona E Puri V Pagano RE Limper AH Macrophage internalization of fungal beta-glucans is not necessary for initiation of related inflammatory responses. Infect Immun (2005) 73:6340–9. 10.1128/IAI.73.10.6340-6349.2005 16177305 PMC1230895

[344] Goodridge HS Reyes CN Becker CA Katsumoto TR Ma J Wolf AJ Activation of the innate immune receptor Dectin-1 upon formation of a ‘phagocytic synapse’. Nature (2011) 472:471–5. 10.1038/nature10071 21525931 PMC3084546

[345] Dillon S Agrawal S Banerjee K Letterio J Denning TL Oswald-Richter K Yeast zymosan, a stimulus for TLR2 and Dectin-1, induces regulatory antigen-presenting cells and immunological tolerance. J Clin Invest (2006) 116(4):916–28. 10.1172/JCI27203 16543948 PMC1401484

[346] Elcombe SE Naqvi S Van Den Bosch MW MacKenzie KF Cianfanelli F Brown GD Dectin-1 regulates IL-10 production via a MSK1/2 and CREB dependent pathway and promotes the induction of regulatory macrophage markers. PLoS One (2013) 8(3):e60086. 10.1371/journal.pone.0060086 23533666 PMC3606242

[347] Schoepfer AM Flogerzi B Seibold-Schmid B Schaffer T Kun JFJ Pittet V Low mannan-binding lectin serum levels are associated with complicated Crohn’s disease and reactivity to oligomannan (ASCA). Am J Gastroenterol (2009) 104:2508–16. 10.1038/ajg.2009.315 19532127

[348] Seibold F Boldt ABW Seibold-Schmid B Schoepfer AM Flogerzi B Müller S Deficiency for mannan-binding lectin is associated with antibodies to *Saccharomyces cerevisiae* in patients with Crohn’s disease and their relatives. Gut (2007) 56:152 10.1136/gut.2006.110007 PMC1856660 17172592

[349] Seibold F Konrad A Flogerzi B Seibold-Schmid B Arni S Jüliger S Genetic variants of the mannan-binding lectin are associated with immune reactivity to mannans in Crohn’s disease. Gastroenterology (2004) 127:1076–84. 10.1053/j.gastro.2004.07.056 15480986

[350] Choteau L Vasseur F Lepretre F Figeac M Gower-Rousseau C Dubuquoy L Polymorphisms in the mannose-binding lectin gene are associated with defective mannose-binding lectin functional activity in Crohn’s disease patients. Sci Rep (2016) 6:29636. 10.1038/srep29636 27404661 PMC4940739

[351] Joossens S Pierik M Rector A Vermeire S Ranst MV Rutgeerts P Mannan binding lectin (MBL) gene polymorphisms are not associated with anti-*Saccharomyces cerevisiae* (ASCA) in patients with Crohn’s disease. Gut (2006) 55:746 10.1136/gut.2005.089136 PMC1856137 16609142

[352] Kim YS Kim Y-H Ye BD Park DW Kim JW Han DS Mannose-binding lectin deficiency is not associated with anti-*Saccharomyces cerevisiae* antibody in Korean Crohn’s disease patients. Clin Chim Acta (2014) 429:206–11. 10.1016/j.cca.2013.12.019 24374090

[353] Underhill DM. Macrophage recognition of zymosan particles. J Endotoxin Res (2003) 9:176–80. 10.1179/096805103125001586 12831459

[354] Katragkou A Pana ZD Perlin DS Kontoyiannis DP Walsh TJ Roilides E. *Exserohilum* infections: review of 48 cases before the 2012 United States outbreak. Med Mycol (2014) 52(4):376–86. 10.1093/mmy/myt030 24682112

[355] Adler A Yaniv I Samra Z Yacobovich J Fisher S Avrahami G *Exserohilum*: an emerging human pathogen. Eur J Clin Microbiol (2006) 25(4):247–53. 10.1007/s10096-006-0093-3 16511679

[356] Sandoval-Denis M Gene J Sutton DA Wiederhold NP Cano-Lira JF Guarro J. New species of *Cladosporium* associated with human and animal infections. Persoonia (2016) 36:281–98. 10.3767/003158516X691951 27616793 PMC4988372

[357] Cheng SC Lin YY Kuo CN Lai LJ. *Cladosporium* keratitis – a case report and literature review. BMC Ophthalmol (2015) 15:106. 10.1186/s12886-015-0092-1 26286482 PMC4545699

[358] Zhou YB Chen P Sun TT Wang XJ Li DM. Acne-like subcutaneous phaeohyphomycosis caused by *Cladosporium cladosporioides*: a rare case report and review of published literatures. Mycopathologia (2016) 181(7–8):567–73. 10.1007/s11046-016-9995-5 27001194

[359] Dixon DM Walsh TJ Merz WG Mcginnis MR Infections due to xylohypha-bantiana (*Cladosporium*, trichoides). Rev Infect Dis (1989) 11(4):515–25. 10.1093/clinids/11.4.515 2672237

[360] Sandoval-Denis M Sutton DA Martin-Vicente A Cano-Lira JF Wiederhold N Guarro J *Cladosporium* species recovered from clinical samples in the United States. J Clin Microbiol (2015) 53(9):2990–3000. 10.1128/JCM.01482-15 26179305 PMC4540897

[361] Mintz-Cole RA Gibson AM Bass SA Budelsky AL Reponen T Hershey GK Dectin-1 and IL-17A suppress murine asthma induced by *Aspergillus versicolor* but not *Cladosporium cladosporioides* due to differences in beta-glucan surface exposure. J Immunol (2012) 189(7):3609–17. 10.4049/jimmunol.1200589 22962686 PMC3470885

[362] Anstead GM Sutton DA Graybill JR. Adiaspiromycosis causing respiratory failure and a review of human infections due to *Emmonsia* and *Chrysosporium* spp. J Clin Microbiol (2012) 50(4):1346–54. 10.1128/JCM.00226-11 22259200 PMC3318518

[363] Hayashi S Naitoh K Matsubara S Nakahara Y Nagasawa Z Tanabe I Pulmonary colonization by *Chrysosporium zonatum* associated with allergic inflammation in an immunocompetent subject. J Clin Microbiol (2002) 40(3):1113–5. 10.1128/JCM.40.3.1113-1115.2002 11880456 PMC120249

[364] Suchonwanit P Chaiyabutr C Vachiramon V. Primary cutaneous *Chrysosporium* infection following ear piercing: a case report. Case Rep Dermatol (2015) 7(2):136–40. 10.1159/000436989 26269703 PMC4519602

[365] Siddiqui AS Zimmerman JL Pulmonary infection secondary to *Chrysosporium zonatum* in an immunocompetent man. Ann Am Thorac Soc (2016) 13(5):757–8. 10.1513/AnnalsATS.201601-083LE 27144802

[366] Warwick A Ferrieri P Burke B Blazar BR Presumptive invasive *Chrysosporium* infection in a bone-marrow transplant recipient. Bone Marrow Transplant (1991) 8(4):319–22. 1756331

[367] Levy FE Larson JT George E Maisel RH. Invasive *Chrysosporium* infection of the nose and paranasal sinuses in an immunocompromised host. Otolaryngol Head Neck Surg (1991) 104(3):384–8. 10.1177/019459989110400317 1902943

[368] Suankratay C Dhissayakamol O Uaprasert N Chindamporn A. Invasive pulmonary infection caused by *Chrysosporium articulatum*: the first case report. Mycoses (2015) 58(1):1–3. 10.1111/myc.12270 25366105

[369] Kenyon EM Russell LH Mcmurray DN Isolation of *Sporothrix schenckii* from potting soil. Mycopathologia (1984) 87(1–2):128 10.1007/BF00436641 6493313

[370] Mehta KIS Sharma NL Kanga AK Mahajan VK Ranjan N Isolation of *Sporothrix schenckii* from the environmental sources of cutaneous sporotrichosis patients in Himachal Pradesh, India: results of a pilot study. Mycoses (2007) 50(6):496–501. 10.1111/j.1439-0507.2007.01411.x 17944713

[371] Barros MBD Paes RD Schubach AO. *Sporothrix schenckii* and sporotrichosis. Clin Microbiol Rev (2011) 24(4):633–54. 10.1128/CMR.00007-11 21976602 PMC3194828

[372] Moreira JA Freitas DF Lamas CC. The impact of sporotrichosis in HIV-infected patients: a systematic review. Infection (2015) 43(3):267–76. 10.1007/s15010-015-0746-1 25701221

[373] Aung AK Spelman DW Thompson PJ. Pulmonary sporotrichosis: an evolving clinical paradigm. Semin Respir Crit Care Med (2015) 36(5):756–66. 10.1055/s-0035-1562901 26398541

[374] Lederer HT Sullivan E Crum-Cianflone NF. Sporotrichosis as an unusual case of osteomyelitis: a case report and review of the literature. Med Mycol Case Rep (2016) 11:31–5. 10.1016/j.mmcr.2016.04.001 27158584 PMC4845149

[375] Vasquez-del-Mercado E Arenas R Padilla-Desgarenes C. Sporotrichosis. Clin Dermatol (2012) 30(4):437–43. 10.1016/j.clindermatol.2011.09.017 22682194

[376] Zhang X Zhang J Huang H Xue R Hu X Li M *Taenia taeniaeformis* in rat favors protracted skin lesions caused by *Sporothrix schenckii* infection: Dectin-1 and IL-17 are dispensable for clearance of this fungus. PLoS One (2012) 7(12):e52514. 10.1371/journal.pone.0052514 23285072 PMC3527553

[377] Guzman-Beltran S Perez-Torres A Coronel-Cruz C Torres-Guerrero H. Phagocytic receptors on macrophages distinguish between different *Sporothrix schenckii* morphotypes. Microbes Infect (2012) 14(12):1093–101. 10.1016/j.micinf.2012.06.001 22771955

[378] Kerscher B Dambuza IM Christofi M Reid DM Yamasaki S Willment JA Signalling through MyD88 drives surface expression of the mycobacterial receptors MCL (Clecsf8, Clec4d) and Mincle (Clec4e) following microbial stimulation. Microbes Infect (2016) 18(7–8):505–9. 10.1016/j.micinf.2016.03.007 27005451 PMC4936759

[379] Longo LV Nakayasu ES Gazos-Lopes F Vallejo MC Matsuo AL Almeida IC Characterization of cell wall lipids from the pathogenic phase of *Paracoccidioides brasiliensis* cultivated in the presence or absence of human plasma. PLoS One (2013) 8(5):e63372. 10.1371/journal.pone.0063372 23691038 PMC3656940

[380] Rizzetto L De Filippo C Cavalieri D. Richness and diversity of mammalian fungal communities shape innate and adaptive immunity in health and disease. Eur J Immunol (2014) 44(11):3166–81. 10.1002/eji.201344403 25257052

[381] Cavalieri D Di Paola M Rizzetto L Tocci N De Filippo C Lionetti P Genomic and phenotypic variation in morphogenetic networks of two *Candida albicans* isolates subtends their different pathogenic potential. Front Immunol (2017) 8:1997 10.3389/fimmu.2017.01997 29403478 PMC5780349

[382] Schonherr FA Sparber F Kirchner FR Guiducci E Trautwein-Weidner K Gladiator A The intraspecies diversity of *C. albicans* triggers qualitatively and temporally distinct host responses that determine the balance between commensalism and pathogenicity. Mucosal Immunol (2017) 10(5):1335–50. 10.1038/mi.2017.2 28176789

[383] Rizzetto L Giovannini G Bromley M Bowyer P Romani L Cavalieri D. Strain dependent variation of immune responses to *A. fumigatus*: definition of pathogenic species. PLoS One (2013) 8(2):e56651. 10.1371/journal.pone.0056651 23441211 PMC3575482

